# Current challenges and opportunities in active and passive data collection for mobile health sensing: a scoping review

**DOI:** 10.1093/jamiaopen/ooaf025

**Published:** 2025-07-18

**Authors:** Christopher Slade, Yinan Sun, Wei Cheng Chao, Chih-Chun Chen, Roberto M Benzo, Peter Washington

**Affiliations:** Department of Information and Computer Science, University of Hawaii, Honolulu, HI 96822, United States; Computer Science Department, Brigham Young University—Hawaii, Laie, HI 96762, United States; Department of Information and Computer Science, University of Hawaii, Honolulu, HI 96822, United States; Computer Science Department, Brigham Young University—Hawaii, Laie, HI 96762, United States; Computer Science Department, Brigham Young University—Hawaii, Laie, HI 96762, United States; Division of Cancer Prevention and Control, Department of Internal Medicine, College of Medicine, The Ohio State University Comprehensive Cancer Center, The Ohio State University Wexner Medical Center, Columbus, OH 43210, United States; Division of Clinical Informatics and Digital Transformation, Department of Medicine, University of California—San Francisco, San Francisco, CA 94143, United States

**Keywords:** mobile health, mHealth, mobile sensing, consumer digital health

## Abstract

**Objective:**

Mobile and ubiquitous devices enable health data collection “in a free-living environment” to support applications such as remote patient monitoring and adaptive digital interventions using machine learning (ML). Despite their potential, significant data collection challenges persist, including issues related to user compliance with reporting data, passive data consistency, and authorization. This scoping review identifies and analyzes these challenges, focusing on barriers to effective data collection.

**Materials and Methods:**

We searched IEEE, ACM, and Web of Science for papers involving training ML models using both active and passive mobile sensing. We used the following search terms: “mobile OR ubiquitous”, “EMA”, “health”, “passive”, and “deep learning OR machine learning”. We only included papers that collected both passive and active data and excluded papers that used a pre-existing dataset.

**Results:**

A total of 77 studies met the inclusion criteria. These studies utilized smartphones, smartwatches, wearable devices, and environmental sensors for data collection. Several studies reported challenges with participant compliance in active data collection, while passive data collection faced data consistency and authorization issues. Efforts to address these challenges were documented in some but not all studies. Using this information, we outline current challenges and corresponding opportunities for data collection in mobile sensing studies.

**Discussion:**

ML techniques can reduce participant burden in active data collection by optimizing prompt timing, auto-filling responses, and minimizing prompt frequency. Simplified interfaces such as user-friendly smartwatch prompts can further improve compliance. For passive data collection, techniques such as optimization of recording times to preserve battery life and motivational techniques to encourage proper device use can increase data consistency.

**Conclusion:**

Mobile sensing offers opportunities for developing intelligent mobile health applications but faces data collection challenges with respect to factors such as compliance, consistency, and authorization. Innovations in ML and user interface design show promise for addressing these barriers.

## Introduction

**Figure 1.**
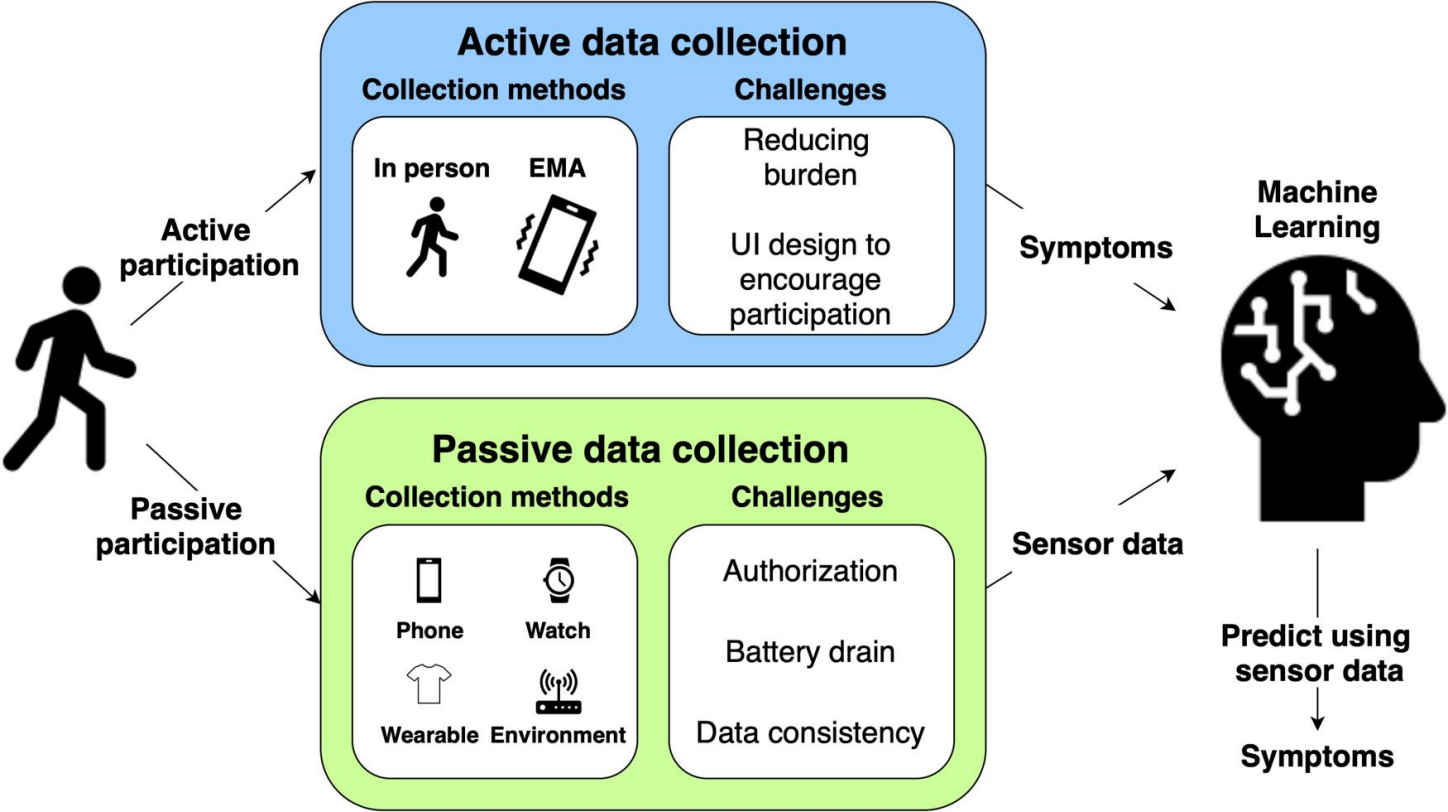
Overview of active and passive data collection methods in mobile sensing research. Active data collection includes EMA or other interventions that require participant attention and engagement, while passive data collection occurs without the need for action from the participant. A common paradigm in the field involves the training of ML models that use passively collected data streams to predict health outcomes that are measured via active data collection methods. Both data collection methods face ongoing challenges, such as minimizing participant burden and ensuring data consistency. This scoping review examines these challenges and offers insights into potential solutions.

In the ever-evolving landscape of healthcare technology, mobile and ubiquitous health sensing systems have emerged as useful tools for real-time health monitoring and intervention. Mobile devices provide opportunities to collect health data “in the free-living environment” rather than in the lab. Traditionally, participants in research studies have been required to actively engage in completing subjective measures related to psychiatry and lifestyle through ecological momentary assessment (EMA). EMA involves “repeated sampling of subjects’ current behaviors and experiences in real-time in the subjects’ natural environment.”^1^ Additionally, consumer devices integrated with various sensors can collect data passively without active participation by the end user.^2^ The combination of active (data collected through EMA and other means that involve the participant) and passive (data collected without the users’ explicit involvement) data streams leads to rich multimodal datasets that can aid researchers in learning nuanced patterns of everyday life.^3^ These rich datasets provide fertile ground for machine learning (ML) to enhance healthcare outcomes^4^^,^^5^ through the use of ML models that are trained using passively recorded data streams as input to predict health outcomes captured by EMA^6–10^ (see Figure 1). The goal of such models is to reduce participant burden in providing active EMA data by predicting the EMA responses using only the passive data. Such models can then lead to the development of applications such as passive remote patient monitoring and adaptive just-in-time digital interventions or therapeutics.

The development of such ML-based adaptive interventions and remote sensing technologies, however, necessitates robust data collection procedures. Despite recent advancements in mobile sensing and ML, significant challenges persist in active and passive data collection on mobile and ubiquitous devices. Stone et al,^11^ through a review of EMA, identified privacy, compliance, and measurement reliability issues. Similarly, Doherty et al^12^ reported challenges concerning compliance, user burden, user engagement, and data validity. Passive data collection also faces obstacles; Boonstra et al^13^ demonstrated that Android and iOS devices completed only 55% and 45% of the passive data collection sessions, respectively, and mobile sensing has been shown to decrease smartphone battery life.^14^ Moreover, creating cross-platform sensing applications for Android and iOS introduces additional complexities.^15^^,^^16^ Other significant challenges include privacy concerns^17^ and usability issues.^18^ These challenges may hinder the efficacy of ML-based adaptive interventions.

This review distinguishes between active and passive sensing to emphasize their unique challenges. Active sensing requires participant engagement, such as completing EMAs to capture subjective or contextual data. This approach often faces participant burden and compliance challenges, resulting in lower data volume. On the other hand, passive sensing continuously collects objective data (eg, heart rate, sleep) through devices without requiring ongoing attention, producing larger datasets but lacking contextual depth. Oftentimes, these data are combined through the use of active data as ground-truth labels and passive data as model inputs in ML-driven continuous monitoring systems,^19–21^ with the goal of obviating the need for active labels after a few are provided.

In this review, we examine mobile health sensing research to understand how logistical and technological challenges can affect the efficacy of remote data collection procedures. Our aim is to expand upon prior reviews that focus on either specific health contexts or a limited array of device types. We include studies that collect passive and active data across various health domains (Figure 1), and we identify strategies that have been used to mitigate or overcome obstacles in mobile sensing data collection. Specifically, we address the following research questions:

**RQ1**: What are challenges in active data collection? What strategies can be implemented to enhance the success of active data collection?

**RQ2**: What are challenges in passive data collection? What strategies have been adopted to improve passive collection rates?

## Related work

Most previous reviews on EMA and mobile sensing have primarily concentrated on specific use cases or diseases rather than on mobile sensing as a general field. Several reviews, for instance, have focused on mental health and digital phenotyping. Seppälä et al^22^ reviewed studies involving mobile phones and wearable sensors for psychiatric disorders and symptoms, Elwirehardja et al^23^ examined sensing studies related to depressive disorders, Benoit et al^24^ focused on schizophrenia and bipolar disorders, and Teepe et al^25^ reviewed depression studies employing just-in-time adaptive mechanisms. Thieme et al^26^ explored the intersection of ML and human-computer interaction (HCI) for mental health. Reviews in the domain of affective computing, where intelligent systems aim to understand human emotions through mobile sensing, have also been conducted.^27–29^ Beyond mental health, Hussain et al^30^ reviewed articles on sleep, Saboor et al^31^ focused on gait, and Stuijt et al^32^ studied mobile sensing in cancer patients. These reviews, however, are limited to specific diseases or disease families rather than providing a broader perspective on mobile sensing methodologies applicable across various healthcare domains. A broader review can reveal cross-cutting strategies that have been successfully applied in one health context and could be adapted to others, fostering interdisciplinary innovation and improving data collection methodologies across diverse applications.

There have been a few prior reviews on disease-agnostic mobile sensing methods, for example by Kumar et al^33^ and Trifan et al,^34^ that provide valuable insights. Kumar et al^33^ conducted a comprehensive literature review to trace the historical evolution of passive sensing and mobile sensing frameworks, addressing aspects such as the adoption and maintenance of sensing software. Trifan et al^34^ and Kulkarni et al^35^ concentrated on smartphone applications. Our contribution extends this body of prior work by reviewing a broader array of data collection vehicles encompassing smartphones, watches, other wearables, and environmental sensors integrated with ML. We uniquely focus on data collection strategies to increase participant compliance and engagement.

## Methods

This scoping review was conducted in accordance with the Joanna Briggs Institute methodological framework for scoping reviews.^36^ The primary author reviewed all search results, while secondary authors independently reviewed a subset to ensure that each paper was assessed by at least 2 reviewers. Disagreements regarding inclusion were resolved by defaulting to inclusion unless exclusion criteria were clearly met. Similarly, data extraction was verified by at least 2 authors to minimize bias.

### Record identification

We identified relevant papers by searching ACM, IEEE, and Web of Science on May 5, 2024, using search terms related to mobile sensing and ML. We selected ACM and IEEE for their extensive computing and HCI literature coverage, and we chose Web of Science for its broad interdisciplinary scope. To ensure our focus was on modern devices, we limited the results to publications from 2016 to the present. The search terms used were as follows: “mobile OR ubiquitous,” “EMA,” “health,” “passive,” and “deep learning OR machine learning”; (full query syntax for ACM: *(mobile OR ubiquitous) AND ema AND health AND passive AND (“machine learning” OR ”deep learning”)* with publication dates limited to 2016 to present; full query syntax for IEEE: *“Full Text Only”:“Mobile” OR “Full Text Only”:“ubiquitous”) AND (“Full Text Only”:ema) AND (“Full Text Only”:health) AND (“Full Text Only”:passive) AND (“Full Text Only”:“machine learning” OR “Full Text Only”:“deep learning”)*). We excluded the term “active” from our query, as it is not commonly used in the context of mobile sensing. Instead, we opted for “EMA” to more accurately capture the relevant studies. We also searched Google Scholar (full query syntax: * (mobile or ubiquitous) AND ema AND health AND passive AND (deep learning or machine learning) (source : ACM OR source : IEEE)*, with a date range from 2016 to present), restricting results to ACM and IEEE publications. Additionally, we searched Web of Science for papers outside of IEEE and ACM venues using the same search terms. However, this search yielded an unmanageable number of results (307 694). To refine the search, we added “adaptive interventions” to the search terms (full query syntax: *ALL = (mobile OR ubiquitous) AND EMA AND health AND passive AND (deep learning OR machine learning) AND (Adaptive Interventions)*, with a date range from 2016 to present) which reduced the results to 303 papers. We choose “adaptive interventions” to include in our review papers focused on adaptive digital interventions that leverage active or passive mobile sensing data to trigger the intervention. We acknowledge that this search strategy likely excluded some relevant papers, but we decided that this is acceptable given the immense number of published mobile sensing papers and the resulting infeasibility of reviewing every possible paper in the field.

The search yielded 774 results. ACM and IEEE returned 167 and 92 results, respectively. Google Scholar added 212 results, and Web of Science returned 303 papers.

### Record selection

We screened the titles and abstracts of the 774 resulting articles to determine their relevance to our focus. We included articles if they utilized mobile or ubiquitous devices to collect mobile sensing data for a health-related purpose. We excluded papers that did not collect both active and passive data. We excluded all review papers and those that did not employ mobile devices for data collection or were unrelated to health issues. This screening process resulted in 280 papers (ACM 89, IEEE 33, Google Scholar 115, and Web of Science 43).

Next, we conducted a full-text review of the remaining 280 papers, excluding those that used previously collected datasets or did not collect both active and passive data. After applying these exclusion criteria, we identified 77 articles for inclusion in our review. The Google Scholar search contributed 46 articles (12 IEEE and 36 ACM). The ACM search yielded 34 articles, including 18 that overlapped with Google Scholar results. The IEEE search provided 8 articles, which also partially overlapped with Google Scholar results by 6 papers. Web of Science contributed 13 additional articles.

The data extraction sheet is organized hierarchically by device type and sorted chronologically within each group. In the literature, “wearables” refer to any device worn by the participant. We categorized wearables into 2 groups: “watches,” which includes devices worn on the wrist, and “other wearables,” which refers to all other wearable devices such as chest monitors. The hierarchy begins with the phone group, which includes all studies utilizing a phone exclusively for data collection. The next level is the watch group, comprising studies that use a watch with or without an accompanying phone. The other wearables group is the third level, which may also involve a watch or phone to supplement data collection. The final and most inclusive group consists of studies employing sensors placed in the environment, such as home or workplace sensors, which may also involve any combination of wearable devices, watches, and phones to supplement the data collection.

### Data extraction

We developed an extraction sheet to systematically gather data from the selected papers (see Table A1 in the Appendix). The sheet includes columns to identify each paper by author and year and to record the following details: disease or health issue addressed, study details (number of participants and study duration), types of active data collected, types of passive data collected, and the analysis and results (including ML and statistical analysis). Additionally, we included a column for details regarding the strategies used for improving active and passive data collection.

## Results

### High-level trends

**Figure 2.**
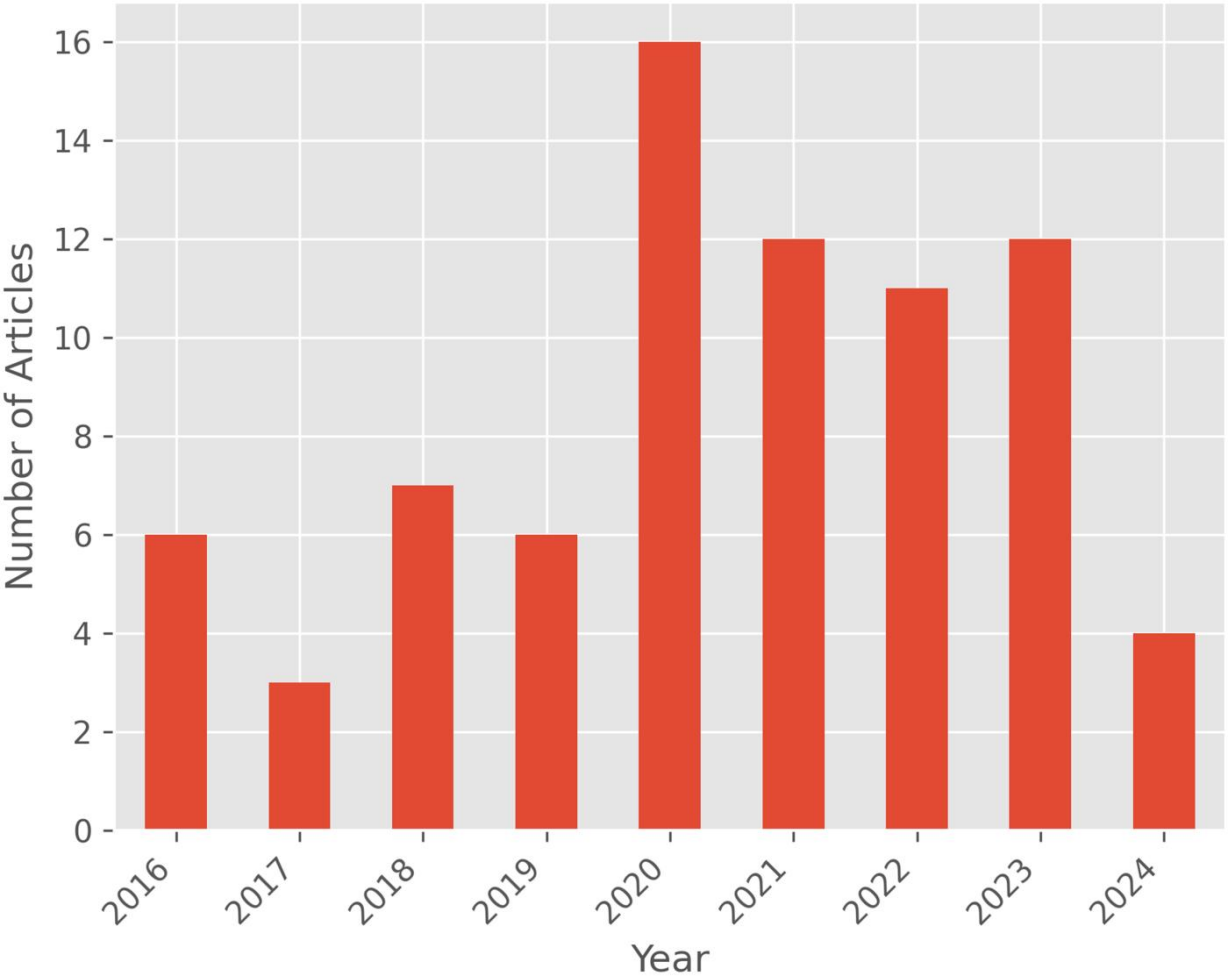
Number of articles per year. The number of mobile sensing studies has generally increased over the years. Note that the data for 2024 include only papers published before May 2024.

**Figure 3.**
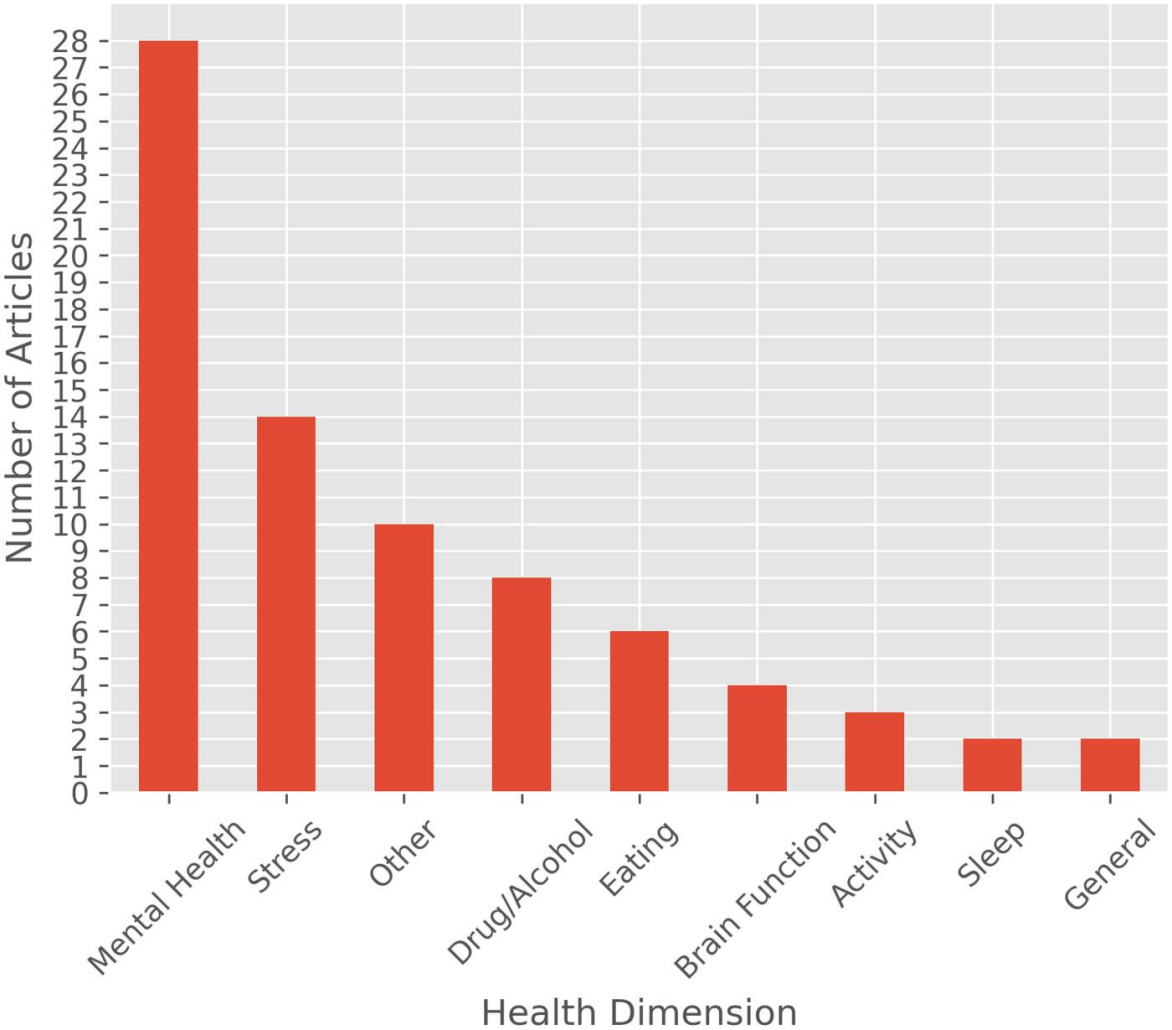
Number of mobile sensing articles by health context. Mental health was the most studied health area involving mobile sensing, especially when including studies on stress. Due to its popularity, we separate studies on stress into a separate category. Health dimensions included in the “other” dimension include chronic pain, hyperactivity, academic performance, couples conflict, heart health, medicine adherence, and multiple sclerosis (n = 77).

The number of published mobile sensing papers continues to rise (see Figure 2), consistent with findings from previous reviews.^34^ Although we did not explicitly exclude any health conditions in our review, mental health emerged as the most frequently studied area (see Figure 3), accounting for over 58% of the studies (including studies focusing on stress). Other categories examined are adjacent to mental health, including drug and alcohol abuse, eating habits, physical activity, sleep, and brain function.

**Figure 4.**
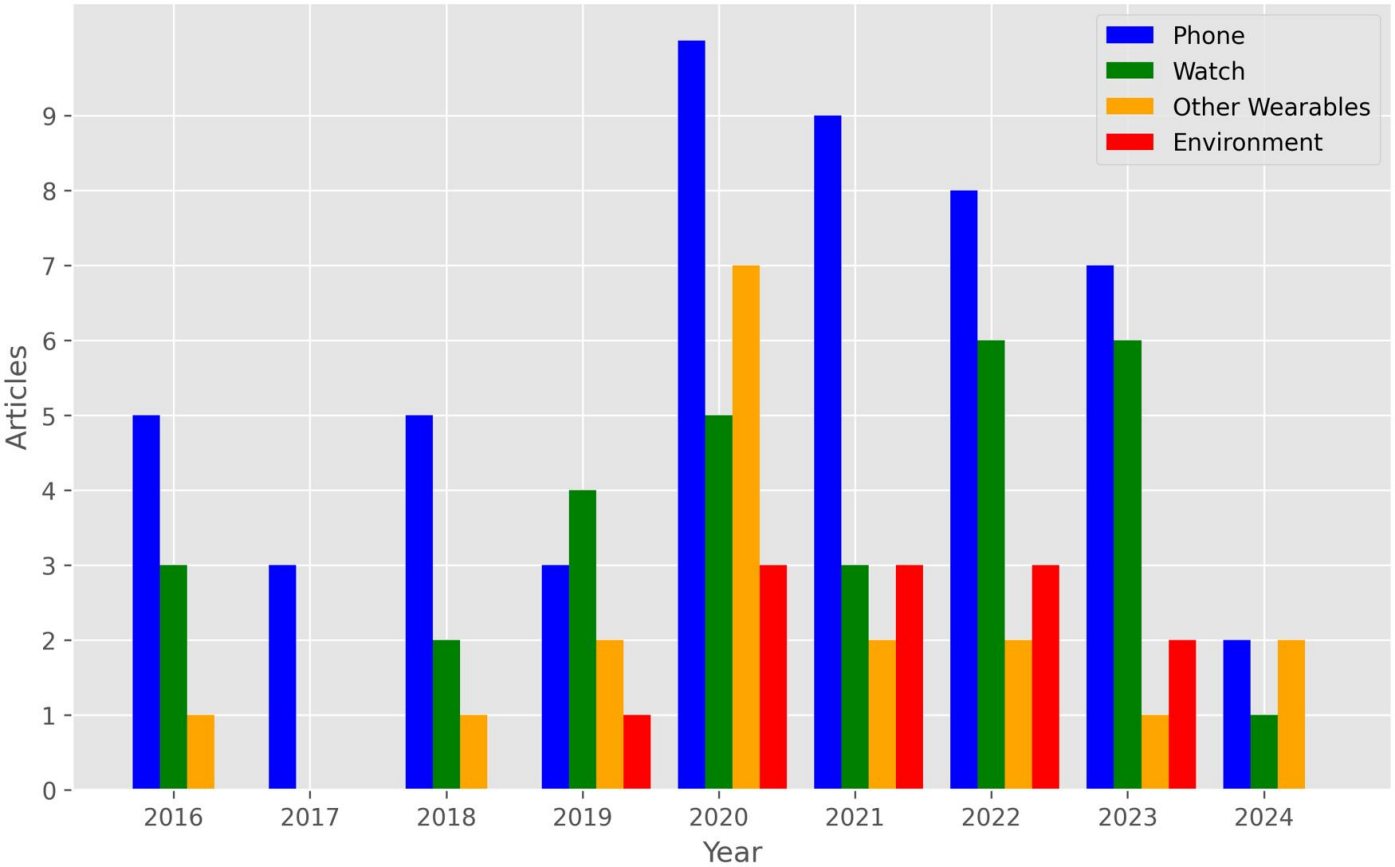
Number of articles by year and device. The number of articles involving off-wrist wearables shows a decreasing trend in more recent years, whereas the number of studies involving watches seem to be increasing. Some articles use multiple categories of devices.

While smartphones have consistently been the primary mobile sensing technology (see Figure 4), the use of wrist-worn devices such as smartwatches has steadily increased. Other non-watch wearables have gained popularity, though recent trends show a decline. This may be due to commercial and clinical-grade smartwatches integrating advanced sensors such as ECG and blood oxygen monitoring, reducing the need for additional devices. For example, while studies commonly used chest straps for heart rate tracking in earlier studies, they now primarily rely on smartwatches for heart rate tracking. Advances in ML methods further decrease the need for extra wearables and environmental sensors by enabling the inference of complex health metrics such as stress and respiratory rate using fewer data sources.

**Figure 5.**
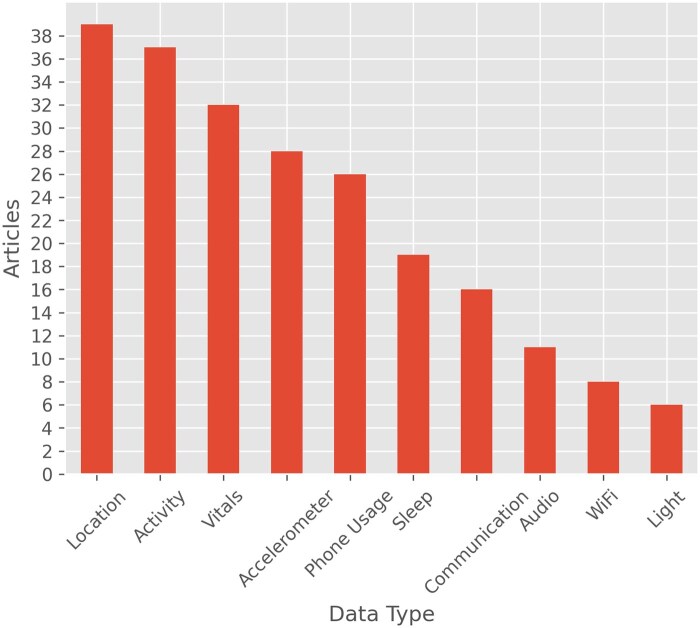
Types of passive data collected. Location, activity, and vitals are among the most commonly collected data types, followed by accelerometer and phone usage. Some papers collected multiple data types.

Location and activity data were the most commonly collected passive data types (Figure 5), followed closely by vitals, accelerometer data, sleep patterns, and phone usage. Vitals were typically collected using watches or other wearables, while location and phone usage data were primarily collected via smartphones. Sleep data had the most varied collection methods, sensed through phone usage, smartwatches, Wi-Fi, and environmental sensors.

Moreover, 56 of the 77 papers collected and reported active data via EMA. Excluding papers that did not perform EMAs or did not report EMA frequency, the average number of EMAs requested per day was 4.89, with a standard deviation of 5.78. One study^37^ requested an EMA every 30 minutes, while another study^38^ only requested an EMA every 2 weeks. Other forms of active data collection included in-person surveys, tests, and videos.

### RQ1: methods to improve active data collection

While reviewing the full text of each paper, we documented any attempts to improve active data collection or manage missing data. One method to enhance EMA compliance mentioned by 2 of the 77 papers^39^^,^^40^ involved linking monetary incentives to the number of completed assessments. Another method to improve compliance, mentioned by 2 of the 77 papers,^41^^,^^42^ consisted of conducting in-person check-ins or having the research team contact participants when missing EMAs were detected. The most prevalent approach to handling missing data, mentioned by at least 6 of the 77 papers, was to ignore time periods with missing data or exclude participants who did not provide sufficient active data.^42–47^ The advantages and disadvantages of these methods have been discussed in other review papers.^11^^,^^12^ Four of the 77 papers^44^^,^^48–50^ used text messages to initiate an EMA instead of application notifications. Text messages may improve EMA compliance rates, as participants might respond more reliably to text notifications—which are generally more prominent than application notifications. Another strategy is allowing participants to select EMA timings. Kim et al^51^ observed that late-night EMA requests were often missed. Jacobson et al^52^ allowed participants to input their waking hours before scheduling EMAs. These methods could improve compliance by adapting the timing of EMAs to the participants’ needs and preferences.

ML was also employed to adapt EMAs to the participant’s needs and preferences. Theilig et al^53^ used ML to adjust EMA timings to be less disruptive. Lustrek et al^54^ developed an ML framework to autofill EMA questions, reducing participant burden. King et al^55^ utilized ML to pre-select EMA questions, minimizing the number of questions required during each EMA. Finally, Arakawa et al^56^ demonstrated that unsupervised learning can reduce or eliminate the need for active data collection. These studies indicate that ML offers a promising avenue for reducing participant burden in active data collection.

HCI innovations can also improve active data collection. Three of the 77 papers^57–59^ provided participants with a user interface that displayed data collection rates, helping motivate users to provide additional data. Kunchay et al^60^ explored using microEMAs, where a single-question EMA could be answered on a smartwatch. Saha et al^61^ examined microEMAs combined with unlock journaling, where participants answered an EMA question to unlock their phones. Additionally, Kim et al^51^ tested the use of explainable AI to facilitate participant self-reflection by explaining how an ML algorithm derived stress levels from passive data, highlighting key features to help participants understand their stress triggers. This integration of ML and HCI presents a promising direction for improving compliance and engagement in active data collection.

### RQ2: strategies to improve passive data collection

Several challenges with passive sensing on smartphones were documented in the reviewed literature. Four of the 77 studies reported issues with participants turning off features or not authorizing data collection,^62–65^ and 4 of the 77 studies exhibited challenges exporting or syncing data to servers,^46^^,^^48^^,^^62^^,^^66^ particularly when executing background tasks on iOS devices. Two studies noted that participants sometimes did not wear the device.^67^^,^^68^ Other challenges included ensuring timestamps were synchronized^69^ and managing changes resulting from mobile operating system updates.^70^

We also identified several methods that improved the reliability of passive data collection. Several studies contacted participants to resolve issues and encouraged them to wear their devices.^66^ This approach can be enhanced by a dashboard for the research team that displays data collection rates.^71^ Hafiz et al^40^ used monetary incentives to encourage participants to wear their devices. Adler et al^46^ utilized EMA to direct participants to start or resume syncing FitBit data, while Biel et al^72^ used EMA to allow participants to fill in missing data. These methods focused on assisting or motivating participants to enhance passive data collection.

In addition to improving passive data collection, several papers discussed ML strategies to handle missing data.^41^^,^^67^ Rashid et al^73^ explored 4 methods of imputing missing data (regression, matrix completion, KNN, and last observation carried forward). Chen et al^41^ used missing compliance/data as a feature to train the ML model. Baglione et al^74^ used ML to activate and deactivate sensors to preserve battery life. These are examples of how ML methods can help mitigate the issue of unreliable passive data collection.

HCI methods can also enhance passive data collection. Kao et al^57^ developed a user interface (UI) that displays data quality to participants, potentially encouraging them to authorize data collection and provide higher quality data. Four of the 77 papers mentioned using Apple’s Health app to improve data collection.^53^^,^^75–77^ The Apple Health app is a central storage location for health data, allowing other mobile applications to read and write data into the app. Since the Health app is integrated into iOS, it offers a more reliable method for data collection. Similarly, Android devices have a centralized health store called Health Connect. These centralized stores could provide a means to enhance passive data collection.

## Key insights and current challenges for the field

### Active data

Several innovations have improved active data collection by reducing participant burden and enhancing compliance. MicroEMAs and unlock journaling can streamline the response process and reduce assessment time,^60^^,^^61^ while ML approaches can optimize EMA timing to minimize disruption,^53^ select the most relevant questions,^55^ autofill responses based on contextual data,^54^ and determine when active data collection can be omitted without compromising model accuracy.^56^ Additionally, several studies have explored using user interface elements, such as EMA progress displays, to motivate continued participation,^57–59^ and Explainable AI has been shown to improve engagement by helping participants understand how their responses contribute to predictions.^51^ An opportunity for future research is to explore how these techniques can be integrated into adaptive EMA frameworks that personalize question selection, timing, and feedback based on individual user behavior and context as well as investigate their impact on long-term engagement and data reliability across diverse populations and health conditions.

Simplified interfaces such as unlock journaling also present potential challenges. Interruptions during phone unlocking may cause participants to provide rushed or inaccurate responses, particularly if the prompt appears at an inconvenient moment.^61^ This inconvenience could affect participant engagement or contribute to additional stress, ultimately impacting data quality. Studies employing unlock journaling have adopted strategies such as adaptive prompt timing or allowing participants to defer prompts to mitigate this. An opportunity for future research is to explore how ML can be leveraged to balance convenience and EMA accuracy by predicting and avoiding disruptive moments for EMA delivery while ensuring robust data collection across diverse populations and health contexts.

While these strategies improve EMA compliance, external factors such as evolving smartphone features and notification management systems introduce new challenges. Features such as notification deferrals and summaries^78^^,^^79^ can reduce the effectiveness of traditional prompts, requiring EMA frameworks to adapt. Context-aware notification systems^80^^,^^81^ and strategic EMA timing^82^ offer potential solutions, but further work is needed to ensure timely responses without increasing cognitive load. Additionally, the integration of Explainable AI into EMA feedback mechanisms warrants further exploration, particularly in facilitating adaptive interventions reinforcing adherence.^51^ An opportunity for future research is to investigate the real-world effectiveness of these approaches, assessing their long-term impact on engagement and data reliability across different health conditions and populations.

### Passive data

Ensuring continuous and reliable passive data collection remains a challenge in mobile sensing studies, particularly due to issues like data loss, inconsistent syncing, and battery consumption. Strategies such as leveraging native mobile OS health stores^53^^,^^75–77^ (eg, Apple Health and Android Health Connect) have repeatedly been used to improve data availability and reliability. Additionally, ML techniques can optimize passive sensing by dynamically adjusting sensor usage based on contextual factors, thereby conserving battery life without compromising data integrity.^74^ EMAs have also been used to alert participants when passive data collection is interrupted, prompting re-engagement to minimize data gaps.^46^ An opportunity for future research is to explore more effective ways to integrate these methods while jointly balancing data quality and utility, user compliance, and energy efficiency across diverse devices and platforms.

Data consistency and authorization challenges also present significant barriers to mobile sensing studies. Missing data due to device non-use, operating system restrictions, or participant burden can reduce model reliability, particularly in mental health and substance use research.^44^^,^^73^ ML-based interpolation and imputation strategies have been used to estimate missing values,^41^^,^^73^ while some studies suggest treating missing data as an informative feature within models.^41^ Additionally, obtaining and maintaining participant consent for continuous passive sensing is a persistent challenge, especially in long-term personal health studies. Approaches such as dynamic consent models, periodic consent renewals, and privacy-preserving data-sharing mechanisms can help ensure participants remain informed and engaged while protecting their data.^17^^,^^20^^,^^46^ An opportunity for future research is to investigate scalable strategies to enhance data integrity and participant trust, particularly in real-world deployments where sensor reliability and user adherence fluctuate over time.

### Limitations

This review spans multiple health contexts, which may limit the depth of analysis for any single condition or domain. Some context-specific details—such as unique data collection challenges or specialized sensing techniques—may not have been fully explored by prioritizing breadth over specificity. While focused reviews on individual health contexts, such as mental health or substance use, have been conducted, this review aims to provide a broader perspective highlighting cross-cutting challenges and strategies applicable across diverse health conditions.

We focused on results that included both active and passive sensing. Consequently, papers that only performed either active or passive sensing were excluded, even though they could have provided additional details regarding data collection methods for mobile sensing. We gleaned valuable insights from a few papers that we are aware of that were excluded by our search and filtering criteria. Chang et al,^83^ for example, did not include active data collection but used another device to provide ground truth labels for ML training. Mishra et al^84^ and Morrison et al^85^ developed an ML model to determine the best time to interrupt users for active data collection, supporting just-in-time adaptive interventions (JITAI). Smets et al^3^ showed that accurately predicting stress and drug craving could be done with only GPS data. Torkamaan and Ziegler^82^ also developed adaptive EMA timings to reduce participant burden and increase usability. Morshed et al^86^ developed an ML model to predict mood without using EMAs.

## Conclusions

We reviewed 77 papers in the field of mobile sensing, analyzing trends across health domains, data collection devices, and methodologies for active and passive data collection. Our findings highlight several challenges with active and passive data collection using consumer devices. We have identified some research opportunities to help address these challenges, including innovations at the intersection of ML and HCI. Further advancements in this field could lead to cost-effective mobile sensing solutions that maximize participant engagement and protocol compliance.

## Supplementary Material

ooaf025_Supplementary_Data

## Data Availability

The data underlying this article are available in the article and in its online supplementary material.
