# Supplementary material for: Current challenges and opportunities in active and passive data collection for mobile health sensing: a scoping review
Source: JAMIA Open. 2025 Jul 18;8(4):ooaf025. doi: 10.1093/jamiaopen/ooaf025 (PMC12274063; doi:10.1093/jamiaopen/ooaf025)
Supplement: ooaf025_Supplementary_Data [file ooaf025_supplementary_data.pdf]

APPENDIX

Table 1. Data extraction sheet for the 77 papers included in the scoping review. For each reviewed paper, we describe the disease studied, study specifics, active and passive data collection methods, and the corresponding analysis and results. The studies are sorted by year of publication in ascending order.[5]

Paper	Disease	Study Details	Active Data	Methods to Improve Active Collection	Passive Data	Methods to Improve Passive Collection	Analysis & Results
			**Phone**				
Aung 2016[89]	Chronic Pain	3 Phase Study: Activity, Speech, Face Detection	EMA: Pain Level via Keppi	Portable device to report pain level (Keppi)	Phone: Physical Activity (GPS, Wi-Fi Location, accelerometer) Emotional State (Audio)	SAINT was developed to assist sensor data collection and signal processing inside phone<br><br>Used pub-sub.	SAINT (Various Models)
Huang 2016[90]	Social Anxiety	18 Participants, undergraduate College psychology Students<br><br>10 Days	In-Person: Brief Psychological questionnaires		Phone: GPS Used Foursquare to identify POI, home, work, food & leisure, and transportation	Used Foursquare to identify POI, home, work, food & leisure, and transportation	Correlation and LR showed significance
Wang 2016[49]	Schizophrenia	48 Participants recruited, 18 dropped out<br><br>1 Year	EMA: 3/Week on Mon., Wed., Fri.<br><br>10 Multiple Choice Questions	Participants are discarded if they have less than 50% EMA responses	Phone: (Android, ChrossCheck App) Activity, Sleep Duration, Sociability, Accelerometer, Light Sensor, Location, App Usage	Days with fewer than 19 hours of sensing are discarded	Bivariate Regression Analysis, GBRT<br><br>Accuracy: Mean Error rate of 7.6%
DeMasi 2017[91]	Well Being	87 Participants, College Students<br><br>8 Weeks	EMA: 4/Day, Mood and Energy<br><br>Intro/Exit Survery	Had limited user participation	Phone: Location	Had incompatibility issues.	SVM, RF, LogR<br><br>Accuracy: 0.75 better than baseline<br><br>Only 33 had enough data for analysis
Saha 2017[63]	Mood Stability	51 Participants<br><br>5 Weeks	EMA: 4/Day (Quedget) Photo to Select Mood	Used Unlock Journaling to collect EMA	Social Media: (Facebook) Linguistic Content of Status updates		Naïve Bayes, LogR, RF, SVM<br><br>Accuracy: 91%
Spanakis 2017[92]	Eating	Study One: 100 Participants: 57 overweight, 43 healthy-weight 2 Weeks Study Two: 100 Overweight 8 Weeks	EMA: Every 2 hours Food Cravings, Emotions	Uses Ecological Momentary Interventions (Includes Assessments and Interventions)	Phone: Location, Activity		Clustering EMI can Improve Eating Habbits (Adaptive Intervention)
Bae 2018[52]	Alcohol Consumption	38 Participants Non-Treatment Seeking, Heavy Drinkers<br><br>28 Days	EMA: 1/Day	Initiated via Text	Phone: (AWARE) Time, Accelerometer, Phone Calls, Text Messages, Keyboard Usage		RF<br><br>Accuracy: 90.9%

Paper	Disease	Study Details	Active Data	Methods to Improve Active Collection	Passive Data	Methods to Improve Passive Collection	Analysis & Results
**Phone**							
Cai 2018 [93]	State Affect (Mood)	220 Participants<br><br>2 Weeks	EMA: 6/Day Once Every 2 Hours Mood		Phone: (Sensus App) Accelerometer, Phone Calls, Text Messages, Location		Statistical Analysis (ANOVA)<br><br>Accuracy: Correlation is significant
Singh 2018 [67]	Risk-taking Propensity	50 Participants (33 male, 17 female)<br><br>10 Weeks	Surveys: Risk Propensity, Demographic, Personality		Phone: GPS, Message, Phone Log (Android smartphone app)	2 participants turned off location	Activity, Reciprocity measure, feed into a OLS regression, Plus various ML algorithms<br><br>Accuracy: Outperforms demography-based models by 39%
Wang 2018 [94]	Schizophrenia	150 Participants<br><br>12 Months	In-Person: 7-Item Brief Psychiatric Rating Scale<br><br>EMA: 3/Week		Phone: (CrossCheck App) Mobility, Sleep, Phone Usage, Phone Calls, Text Messages, Ambient Light, Ambient Sound		GBRT<br><br>Accuracy: MAE +/- 1.45 with EMA +/- 1.59 w/o EMA
Zhou 2018 [79]	Physical Activity	64 Participants Control: 10,00 Step Goal<br><br>Intervention: Adaptive Step Goal<br><br>10 Weeks	Step Goal via Notification		Phone: (iOS) Steps	Data Collected via HealthKit	Behavior Analytics Algorithm determined goal<br><br>Results: 95% Less Step Reduction
Zhang 2019 [66]	Mood Instability	86 Students Cohort 1: 42 Students 1 month. Cohort 2: 44 Participants 2 weeks. 68 participated 2 weeks, 18 dropped out	EMA: 3/Day Ekman's Discrete Mood Survey		Phone: Accelerometer, Microphone, Audio, Light, Screen Status, WiFi, App Usage	Background thread ran every 10 minutes. Some participants switched off or unauthorized, which were removed from data	SVM, MLP, MVDS, CDMF<br><br>Accuracy: 78.33% F1: 77.78%
Etienne 2020 [95]	Stress	12 Participants College Students<br><br>2 Weeks	EMA: 3/Day Stress Level		Phone: Accelerometer, Light, Audio, Location		Transfer Learning, SVM<br><br>Accuracy: 90%
Jacobson 2020 [54]	Depression	31 Participants<br><br>7 Days	EMA: 1/Hour<br><br>Mood and Heart Rate Assessment	Participants pre-reported awake hours	Phone: Location, Heart Rate, Phone Calls, Weather		XGB, RF<br><br>Accuracy: CI of 0.587
Rhim 2020 [68]	Subjective Well-being	78 college students<br><br>4 Months	EMA: 1/Daily Life Satisfaction, Emotional Experience		Phone: Activity, GPS, App Usage, and Screen Status	Contacted participants when data was missing to resume collection	Hierarchical Regression DT, RF<br><br>Accuracy: Up to F1: 70%
Ameko 2020 [96]	Emotion Regulation	110 Participants 81 Female 29 Male 4 Excluded<br><br>5 Weeks	EMA: 6/Day Social, Interaction, Anxiety Level<br><br>MetricWire Mobile App	Reminder notifications are sent if not completed in 30 minutes and removed if not completed in 45 minutes	Phones: Activity and GPS		LogR and T-Tests<br><br>Accuracy: Significant

Paper	Disease	Study Details	Active Data	Methods to Improve Active Collection	Passive Data	Methods to Improve Passive Collection	Analysis & Results
**Phone**							
Obuchi 2020[97]	Brain Functional Connectivity	105 Participants 1st Year Students 79 Days	In-Person: fMRI		Phone: (NeuroSence App) Location, Activity, Speech, Sleep	Used on-device pre-trained model to detect speech to preserve privacy	SVM<br><br>Accuracy: F1 0.793
Rashid 2020[75]	Social Anxiety	80 Participants with Social Anxiety<br><br>5 Weeks	EMA: 1/Day at 10PM for two Hours Subject Measures of Anxiety		Phone: (MetricWire) GPS, Pedometer, Accelerometer, Acitivity, Phone Calls, Text Messages	Method to impute missing values: 1 MICE: regression, 2 Matrix Completion, 3KNN, 4 Last Observation Carried Forward	RF, MERF, CatBoost<br><br>Accuracy: Imputation of missing values reduces error 22%
Buda 2021[64]	Daily Happiness	303 Paticipants Included Cohort 1: 221 users from 5 different countries 2nd Cohort: 481 college students<br><br>2-3 Weeks	EMA: 5/Day Momentary Happiness Daily Happiness question (Likert)		Phone: Usage Pedometer, GPS, Ambient Light, Noise Level, Battery Level, Lock/Unlock, Phone Calls	Users could opt out of certain types of data collection<br><br>iOS users were dropped from the results due to lower numbers and inconsistent data collection	LogR, RF, Multilayer Perception and XGB<br><br>Accuracy: 13% improvement in AUC and 27% in F-score
Dong 2021[98]	Influenza Detection	448 Participants 24 States<br><br>1 year	EMA: 1/Day Report Flu like symptoms		Phone: (ReadiSens) GPS, Wifi, Mobility, Social Interactions, App Usage, Activity	2700 Started, on 448 had 14 days of data.	GNN, LogR, SVM,RF, XGB, MLP<br><br>Accuracy: F1 Score 0.8762 on GNN
Dong 2021[99]	Mental Health	2700 Participants<br><br>Collection period over a year, 14 Days required	EMA: Every 12 Days to Evaluate Mental Health		Phone: (ReadiSens) GPS, Wifi, Mobility, Social Interactions, App Usage	Developed GNN to deal with low number of labels	Graph Instance Transformer<br><br>Accuracy: Beat baseline by 8.8%
Meegahapola 2021[100]	Eating	84 Participants College students in Mexico<br><br>60 Days	EMA: 3/Day<br><br>Food intake self-reports: Eating Episodes, Food categories<br><br>Concurrent Activities, Stress Level, etc.	Sent EMA after eating episodes to not interrupt and modify users	Phone: App Usage, Accelerometer, Battery Events, Screen Events, Location		Pearson and Point-Biserial correlation, RF, NB, GBM, NN, XGB, AdaBoost, SVM<br><br>Accuracy: 83.49% - 87.81%
Srikanthan 2021[65]	Traumatic Brain Injuries	375 Participants, 17 with TBI<br><br>Recruited through Ads<br><br>12 Weeks	EMA: Daily Health Questions		Phone (iOS): Accelerometer, gyroscope, magnetometer, pressure, altitude, battery, pedometer, accessibility features<br><br>Sensor data polled every second	Users had the option to turn off polling<br><br>Users with insufficient data were not included in results	DEC, XGB Outlier Detection<br><br>Accuracy: 88% and Sensitivity of 74%

Paper	Disease	Study Details	Active Data	Methods to Improve Active Collection	Passive Data	Methods to Improve Passive Collection	Analysis & Results
**Phone**							
Kim 2022[53]	Stress	36 Participants College Students<br><br>25 Day<br>10 Day Model<br>15 Day Prediction	EMA: 4/Day at 4 Hour Intervals Stress level	11 PM Was too late for EMA.	Phone: (MindScope) Accelerometer, Activity, GPS, App Usage, Noise Levels	Users Provide Common Locations Explainable AI (XAI) Reports to User Reasons Why Stress is High (Location, Noise, etc). Provides Stress Intervention Ideas	Explainable AI, XGB Algorithm Used<br><br>Statistical Analysis
Meegahapola 2021[101]	Alcohol Consumption	241 Participants Young Adults in Switzerland<br><br>3 Months, Weekends only	EMA: Hourly from 8PM-4AM<br><br>Log Drinks		Phone: (Android) Accelerometer, Battery, Bluetooth, Location, Wifi, App Usage, Screen Usage, Proximity	More details in additional papers<br><br>Data separated by continuous sensing and interaction sensing	Pearson, and Point-biserial correlation<br><br>Accuracy: 75%-86%
Meegahapola 2022[102]	Mood	678 Participants Eight Countries	EMA: 20/Day (2 Hours to Complete)<br><br>Start/End of Day		Phone: (Android) Steps, Location, Wifi App Usage, Screen Events, Phone Usage, Activity, Ambient Light, Notifications		Built both population and personalized (hybrid) models<br><br>Models for all participants, and each country<br><br>Accuracy: AUROC-score 0.78-0.98 and 0.76-0.94
Wang 2022[73]	Mental Health of First-Gen College Students	80 Participants 27 First-Generation Students<br><br>1 Year	EMA: Weekly Mental Health: Depression and Anxiety (PHQ-4)		Phone: Activity, GPS, Phone Usage, Locks/Unlocks, Sleep Regularity<br><br>Used iOS and Android API to Recognize Activity	Used App Center to monitor and update smartphone app. iOS used VoIP push alerts to run background tasks. Had dashboard to monitor collection activity	LogR, RF, GBM. LSTM, sub-NN.<br><br>Accuracy: F1-0.71 (Best Deep learning)
Bae 2023[103]	Alcohol Consumption	75 Participants With Risky Behavior<br><br>14 Weeks	EMA: 2/Week<br><br>Report Alcohol Consumption	SMS used to initiate EMA	Phone: (AWARE) Accelerometer, Location, Wifi, Gyroscope, App Usage, SMS, Ambient Light, Battery	Most informative features: Time of Day, GPS	XGB<br><br>Accuracy: 95%
Bardram 2023[78]	Type 2 Diabetes	12 Participants<br><br>6 Weeks<br><br>Feasibility Study	EMA: Blood Glucose, Smoking, Weight, Alcohol, Lifestyle, Emotional distress, well-being, etc.		Phone: Battery, Screen, Noise, Location, Activity, Weather, Steps	Mentions event-based vs. polled-based collection. Health App (iOS) could have improved collection of Blood Glucose	Analyzed the usability and feasibility of the app. No ML analysis

**Paper**	**Disease**	**Study Details**	**Active Data**	**Methods to Improve Active Collection**	**Passive Data**	**Methods to Improve Passive Collection**	**Analysis & Results**
**Phone**							
Lustrek 2023[56]<br><br>GS/ACM	Stress	Proposes design of JITAI, but does not include a study	EMA: Diaries	Uses AI to automatically fill in parts of the EMA	Phone: Detect context and stress from passively collected data		AI helps users complete EMA and detects context and stress levels<br><br>Adaptively suggests exercises to reduce stress
Ning 2023[104]	Mood Disorders (Brain Health)	85 Participants<br><br>4-5 Weeks	In-Person: 2 Cognitive Assessments<br><br>14 Days Apart		Phone: (BiAffect Keyboard) Keyboard Usage	Used Keyboard Interactions as a passive sensing stream	Statistical Analysis<br><br>Diurnal Differences and Typing difference showed differences
Vandelanotte 2023[105]	Physical Activity	Proposes design of JITAI but does not include a study.	Q&A: Physical Activity Related Question to proposed to generative	AI Uses generative AI as means of active data collection	Phone: GPS, Location, Weather	Use location to determine weather and adapt "Nudges"	Proposal Only
Wang 2023[106]	Auditory Verbal Hallucinations	384 Participants<br><br>30 Days	EMA: 4/Day<br><br>Audio Diary		Phone: (Android) GPS, Lock/Unlock, Phone Usage, Activity, Ambient Light, Sleep		Deep Learning (RNN)<br><br>Accuracy: F1: 0.78 (Voice Diary Only)
Nepal 2024[72]	Mental Health	215 Participants<br><br>Students at Dartmouth College<br><br>123 Completed to Graduation<br><br>4 Years	EMA: Weekly<br><br>Anxiety and Depression	75% Completeness Score	Phone: Activity, Locations, Phone Usage, Audio Play, Sleep	90% Completeness Score 20% Location Missing, 12% Audio, 22% Steps<br><br>Updates Cause Challenges in Longitudinal Studies<br><br>Android introduces changes in background tasks	Summary Analysis

**Paper**	**Disease**	**Study Details**	**Active Data**	**Methods to Improve Active Collection**	**Passive Data**	**Methods to Improve Passive Collection**	**Analysis & Results**
**Watch**							
Gjoreski 2016[107]	Stress	5 Participants	EMA: Random Periods<br><br>Stress Level		Watch: (Empatica) Activity, GSR HR, BVR		Accuracy: 92%
Biel 2018[74]	Eating	122 participants (18-26 years old)<br><br>10 Weekdays	EMA: Food Intake with Photos, Where, With Whom<br><br>EMA: End of Day Survey	Scheduled Notifications at mealtimes, end of day survey	Watch (FitBit): Activity Data	End of Day survey to fill in missing FitBit Data	RF<br><br>Accuracy: 84%

**Paper**	**Disease**	**Study Details**	**Active Data**	**Methods to Improve Active Collection**	**Passive Data**	**Methods to Improve Passive Collection**	**Analysis & Results**
				**Watch**			
Sefidgar 2019[108]	Discrimination Events	209 Participants 1st Year College Students<br><br>6 Months (2-Terms)	EMA: 2/Week Discrimination Event<br><br>4/Week for 2 weeks		Phone: (AWARE) Location, Screen Status, Call Logs, Activity<br><br>Watch: (FitBit Flex 2) Steps, Sleep	Did not use FitBit and Phone all the Time	LR, Statistical Analysis<br><br>Accuracy: N/A
Theilig 2019[55]	Mental Health	6 Users<br><br>29 Days	Personal Surveys: Daily Activities	EMA to test receptivity<br><br>Time influenced by ML Model	Watch: Activity Data	Read Health Data from HealthKit (iOS), or watch app<br><br>Includes JITAI feasibility	ML model predicted the best time to send EMA about mental health from Activity Schedule (LSTM)<br><br>Accuracy: 58% Activity Recognition
Hafiz 2020[42]	Bipolar Disorder	15 Participants 9 healthy 6 with DB<br><br>7 Days	EMA: (FitBit) 1/Day 3 Cognitive Tests (Choice Reaction Time, N-back, Stoop)	Used watch alarms as reminders<br><br>Monetary Incentive Paid per test taken	Watch (FitBit): GPS, Activity, Sleep	Paid per night watch was worn	KNN, SVM, RF, XGB<br><br>Accuracy: 74% (KNN)
Kunchay 2020[62]	Alcohol and Marijuana Consumption	1 Participant<br><br>1 Week	EMA: 5/Day Details of Substance Use	Used microEMA on Watch to reduce burden.	Phone: (AWARE) Accelerometer, Gyroscope, App Use, Noise, Batter, Phone Calls, Text Messages, Location, WiFi, Weather<br><br>Watch: (HealthKit) Steps, Stand Hours, Activity, Heart Rate, Calories, Sleep		Feasibility Study With Only 1 Participant
Adler 2021[48]	Stress	775 Participants<br><br>14 Months<br><br>During Medical Internship	EMA: Daily Mood (Likert Scale)	Needed to filter participants with low data quality	Watch: (FitBit Charge) Heart Rate, Steps, Sleep	Used EMA to help ensure FitBit Data synced to secure storage. Dropped hours without data	Generalized Estimating Equations
Bonaquist 2021[109]	Mental Health	40 Participants Adolescents	EMA: (REDCap)<br><br>1/week PHQ-9		Phone: (AWARE) GPS, Accelerometer, Phone Calls, Wifi, Phone Usage<br><br>Watch: (FitBit) No details included		XGB, XGBRF, RF, ExtraTrees, GBM, AdaBoost, Light GBM, Catboost<br><br>Accuracy: RMSE 5.3 (XGBRF)

Paper	Disease	Study Details	Active Data	Methods to Improve Active Collection	Passive Data	Methods to Improve Passive Collection	Analysis & Results
**Watch**							
Lee 2022[61]	Sleep	Preliminary: 136 Participants (University) and 54 (Major Airline)<br><br>Initial Deployment: 11 Participants, 2 Weeks<br><br>Deployment: 20 Participants, 8 Weeks	Initial Deployment: Online: Daily Sleep Survey<br><br>Deployment: Online: 2/Day	Presents a Dashboard to User about progress and suggestions<br><br>Overall compliance rate from 0.46 to 0.58	Watch: (FitBit) Fitness, Bedtime, Wake Time, Heart Rate, Steps<br><br>Calendar: (Google) Updates	Implements JITAI for sleep. Adjusts for missed guidance	
Tsai 2022[40]	Panic Attacks	59 Participants with Panic Disorder<br><br>1 Year	In-Person: Panic Disorder Severity Screening<br><br>EMA: Every 2 Weeks Anxiety Levels		Phone: Physiological Data<br><br>Watch: (Garmin or FitBit) Steps, Heart Rate, Activity		RF, DT, Linear Discriminant Anaylysis, Adaptive Boosting, XGB, Regularized Greedy Forests<br><br>Accuracy: RF 67.4%-81.3%
Velmovitsky 2022[77]	Stress	1 Participant (Prosposal)	EMA: */Day Stress		Phone: (iOS HealthKit)<br><br>Watch: (Apple) ECG (Empatica E4) HRV	Used iOS HealthKit to collect passive data	RF, SVM<br><br>Accuracy: 85% (RF) 70% (SVM)
Wang 2023[110]	Social Anxiety	46 Participants College Students<br><br>During Social Conversations Through Video Calls	Online: Post Survey		Watch: (Empatica E4) Heart Rate, EDA, Accelerometer, Temperature		kNN, SVM, NB, Tree, AB, RF, MLP<br><br>Accuracy: Best (MLP) F1: 0.69
Arakawa 2023[58]	Hyperactivity in Children	61 Children	25 with hyperactivity Parents provided labels on hyperactivity	ML models originally used parent labels, but later used contextualized data<br><br>85% vs 82%.	Watch: (Apple Watch) Custom App: Heart Rate, Location, Bluetooth and Accelerometer	Apple's Library provided best data collection results for accelerometer	SGD, DT, RF, GB<br><br>Accuracy: 85.2%
Can 2023[47]	Mood and Emotion	14 Participants<br><br>7 Days (Not Consecutive)	EMA: 1/3 Hour Session Perceived Emotion (7 Likert Questions)<br><br>Initiated by emails	Passive data was discarded when participants missed an EMA	Watch: (Empatica E4) Accelerometer, Heart Rate, Temperature, EDA		RF, DT, kNN, MLP, LR, and SVM<br><br>LSTM and Bidirectional LSTM provided best results<br><br>Accuracy: 95%
Toshnazarov 2024[111]	Stress	Lab: 26 Participants<br><br>Real Life: 18 Participants 2 Weeks	EMA: 12/Day<br><br>Stress Dynamics		Phone: Activity, Phone Calls, Screen State, Location<br><br>Watch (Samsung) HR	Used an always running foreground service (Android)	Accuracy: F1 0.84 Lab, 0.71 Real Life

**Paper**	**Disease**	**Study Details**	**Active Data**	**Methods to Improve Active Collection**	**Passive Data**	**Methods to Improve Passive Collection**	**Analysis & Results**
				**Other Wearables**			
Chatterjee 2016 [112]	Smoking Cessation	61 Participants<br><br>24 Hours Before Quit Date, 72 Hours After Quit Date	EMA: Cravings and Physical Activity	Daily in-person check-in to verify data	Phone: GPS<br><br>Watches: Accelerometer and Gyroscope<br><br>Wearable: (AutoSense): ECG, Accelerometer, Respiration.	improve	Developed mCrave model<br><br>Accuracy: 72%
Sarker 2016 [113]	Stress	38 Participants	EMA: 5.8/day<br><br>Stress, Drugs, and Smoking		Phone: Location, Accelerometer<br><br>Wearable: Breathing Rate, ECG, HRV		Accuracy: F1 score 0.71
King 2019 [57]	Stress in Pregnancy	18 Women Not Pregnant 16 Lab Activities<br><br>Real World: 18 Pregnant Women 2 Days	EMA: Hourly for 12 Hours<br><br>Stress Test	Developed a ML Model to determine which EMA questions to use, prior to a study, to help reduce EMA burden	Wearable: (Biostamp) ECG, (Polar H7) Heart Rate (Neurlog GSR), GSR/Skin Conductivity Only Biostamp in real world study.		SVM, DT, Ada Boost, NB, NN<br><br>WorriedStress was the best EMA question to use
Delmastro 2020 [114]	Stress detection with mild cognitive impairment	9 Older adults, 5 women, 4 men	In-person tests: SCWT	Data collected in lab	Wearables: (Bio Harness) Heart Rate, HRV, EDA, ECG<br><br>(Shimmer3) EDA	Data collected in lab	BN, SVM, k-NN, c4.5 DT, RF AB<br><br>Accuracy: 80%-95.8%
Chatterjee 2020 [44]	Smoking “opportunity contexts”	126 Smokers 55 Female 71 Male<br><br>4 Days Pre-Quit, 10 Days Post-Quit	EMA: 12/Day Cigarette Availability and Smoking Allowance	67.2% compliance<br><br>36 did not provide enough data to be included	Wearable: (Chest and wristband) ECG, Respiration, Accelerometer<br><br>During Waking Hours	Preprocessed data to account for noisy data	RF, SVM-RBF, LogR, Adaboost classifiers<br><br>Accuracy: F1: 74.3%
Gupta 2020 [115]	Couple Conflict	87 Couples<br><br>18-25 Years old<br><br>1 day	EMA: Hourly (9AM-12PM)<br><br>Mood and Quality of Interactions		Phone: GPS 3-minute audio sample every 12 minutes<br><br>Watch: EDA (Q sensor)<br><br>Wearable: ECG, (Actiwave)	Participants were provided a smartphone for the 1-day study	Subpopulation clusters, K-means algorithm and FNN<br><br>Accuracy: F1-76%
Mishra 2020 [86]	Stress	27 Participants<br><br>3 Days	EMA: Every 30 minutes, for 8 hours per day<br><br>Stress level (1-5)	Collected on Watch<br><br>Participants can self report	Watch: (Amulet) Accelerometer, HRV, Activity<br><br>Wearable: (Polar H7)		SVM, RF<br><br>Accuracy: F1-score: In field 0.72 (0.66 without activity)

Paper	Disease	Study Details	Active Data	Methods to Improve Active Collection	Passive Data	Methods to Improve Passive Collection	Analysis & Results
**Other Wearables**							
Zhang 2020[71]	Eating	20 Participants<br><br>10 Semi-free-living Study<br>10 Free-living Study<br><br>11 with 9 w/o Obesity<br><br>420 Total Hours Collected	Video	Used Video to provide input labels<br><br>Participants could delete video	Wearable: (Necklace) IMU, Proximity, Ambient Light<br><br>Detects Eating Episodes	Need to ensure devices time is correct	GBM Classifier<br><br>Accuracy: F1 81.6% and 77.1%
Elvitigala 2021[116]	Stress	10 Participants Office Workers<br><br>4 Week	In-Person: Weekly, Rate Agreement with Detected Stress	Allowed Users to Select Interventions	Phone: Calendar Integration<br><br>Wearable: (Inertial Measuring Unit Sensor Tag), Mount on Shoe		Linear-Discriminant Analysis ML Model
Kumar 2022[60]	Ambulatory Arrhythmia	24 Participants<br><br>2 weeks	EMA: Daily Stress Level, Sleep, Dietary Details<br><br>Any Time: Self-Report of Symptoms	UI included visual display of both active and passive data collection rates<br><br>Helped participants fix collection errors	Phone: Activity, Location, Sleep, Weather, Noise Level<br><br>Wearable: (EcgMove4): ECG HR, HRV, and MET		Only analyzed the feasibility of the system
Ullah 2022[117]	Smoking	92 Participants	EMA: 12/Day (3 per 4 Hour Block)		Phone: GPS Chest band: ECG, Respiration<br><br>Watch: (both Arms) Accelerometer/gyroscope	Used Event-based encoding to reduce noise	Deep learning model (LSTM)<br><br>Includes historical influence and recent<br><br>Accuracy: 85% of lapses
Daryabeygi-Khotbehsara 2024[118]<br><br>WofS	Activity Recognition	15 Participants	In-Person: Cameras to Record Activity		Wearable: (SORD) Accelerometer, Gyroscope (ActivPal) Detects Standing, Walking, Lying, and Sitting	Compared Devices	Deep Learning<br><br>Accuracy: Above 95%
King 2024[51]	Emotional State	45 Participants<br><br>10 Days	EMA: 10/Day Access Mood, 13 Questions, 9 negative, 4 positive	Initiated Via Text Message, Paid per response	Watch: EDA, Temperature, Accelerometer<br><br>Wearable: (Chest Patch) ECG, Accelerometer		k-means Clustering, RF and NN<br><br>Accuracy: Descrease negative effect by >3 points
Presseller 2024[41]	Binge-Spectrum Eating Disorders	22 Participants<br><br>2 Weeks	EMA: Self-Initiated Reports	Used high incentives ($500) for compliance<br><br>Participants were monitored and reminded	Wearable: (Dexcom G6 CGM) Blood Glucose		RF<br><br>Accuracy: 82% for Meals, 78% Non-Meals

Paper	Disease	Study Details	Active Data	Methods to Improve Active Collection	Passive Data	Methods to Improve Passive Collection	Analysis & Results
**Environment Sensors**							
Diethe 2018[119]	General Health	30 Homes	EMA: Questionnaires		Home Sensors: Temperature, Humidity, Noise Level, Luminosity Video<br><br>Wearable: Accelerometer	Actors act out a script to create labels<br><br>Unsupervised learning<br><br>Post-Hoc analysis of data	Discussed Methods<br><br>LR, Unsupervised approaches as well<br><br>Accuracy: Not reported
Chen 2019[43]	Moderate Cognitive Impairment and Dementia	119 Participants<br><br>82 Healthy 32 MCI or Dementia<br><br>12 Weeks	EMA: Daily, 2-Questions, Bi-Weekly Assessment Test (iPhone app)	Participants contacted directly to use devices when not collecting data	iPhone: Phone Usage, Messages, Unlocks, App History<br><br>Apple Watch: Heart Rate, Breathing<br><br>Beddit: Sleep Interpolation for missing Heart Rate. Non-compliance used as a feature		EXGB<br><br>Accuracy: AUROC = 0.8
Tong 2019[45]	Multiple Sclerosis (Fatigue and Health State)	198 Patients<br><br>6 Months	EMA: Weekly Surveys, Daily Questions	24 patients did not complete surveys<br><br>Only 73 attempted daily questions	Watch: Sleep and Activities<br><br>Scale: Weight<br><br>Sleep Tracker: Sleep		Regression Model<br><br>Accuracy: MAE ranges from 0.58, 1.0, 0.093, 0.097 to predict scores
Baglione 2020[76]	Medication Adherence	Study Protocol, No Participants	EMA: 1/Day Stress Ratings		Phone: Acceleration, Location, Sound, Proximity, Communications, App Usage, Light Level<br><br>Wearables: Heart Rate GSR,ECG, Activity<br><br>Beacons: Proximity, Temperature	Use ML and context to turn off sensors to improve energy efficient sensing	Develop personalized models to adapt to various patients
Goldstein 2020[120]	Weight Control	121 Participants Overweight<br><br>10 Weeks	EMA: 6/Day<br><br>Eating Relapses and Causes	EMA determined cause	Scale: Weight		DT<br><br>Accuracy: 79.8% Lapse Prediction

**Paper**	**Disease**	**Study Details**	**Active Data**	**Methods to Improve Active Collection**	**Passive Data**	**Methods to Improve Passive Collection**	**Analysis & Results**
**Environment Sensors**							
Kao 2020 [59]	General Health	212 Health Workers<br><br>3 Cohorts<br><br>10 Weeks	EMA: 1st Daily job, health, personality<br><br>2nd Daily - Psychological Flexibility or Capital	Participants could monitor compliance rate in app	Fitbit: Heart Rate, Steps, Sleep, Activity<br><br>OMSignal Garment: ECG, breathing, motion<br><br>Unihertz Jelly Pro: Accostic Data<br><br>Phone: Phone Usage, Battery Life, Screen time<br><br>Hospital Sensors: Location in Hospital, Light, Temperature, Motion	Participants could monitor their data quality in the app	Radial Basis function. Personalized models used as well as a baseline model<br><br>Accuracy: Better than baseline w/o historical data
Cook 2021 [70]	Clinical Scores related to Brain Function, Mobility, and Verbal Ability	21 Participants, > 45 Years Old<br><br>1 Month	EMA: Functioning, Socialization, Activity, Fatigue and Mood<br><br>In-Person: Clinical Assessments		Watch: Location, Accelerometer, Gyroscope<br><br>Home: Infrared Motion/Light, Temperature, Door	Used two watches to collect data continuously<br><br>Still missing time periods that was filled in with RF regression	GBM<br><br>Accuracy: Best 0.881
Saha 2021 [121]	Psychological Constructs	754 Participants<br><br>1 year	During Enrollment: Cognitive Ability, Personality Traits, Anxiety, Well-Being Tests		Phone: Physical Activity, Mobility, Phone usage<br><br>Watch: (Garmin) Step Count, Sleep<br><br>Bluetooth Beacons: Desk Use<br><br>Social Media: Facebook	Includes several different devices and social media	Compares general models, clustering, and personal models<br><br>Generalized: Better for cognitive ability<br><br>Contextualized: Better for personality traits, anxiety, and sleep quality
Shin 2021 [122]	Stress	50 Participants<br><br>In-Lab Study	In-Person: Self Report		Watch: (Empatica E4, MetamotionR) HR, HRV<br><br>Wearable: (Zephyr Bioharness) Motion<br><br>Environment: (Radar) Reflected Radar Signals		Accuracy: 76.22% (11.57% better than wearables)

Paper	Disease	Study Details	Active Data	Methods to Improve Active Collection	Passive Data	Methods to Improve Passive Collection	Analysis & Results
**Environment Sensors**							
Booth 2022[46]	Stress	606 Participants<br><br>Full-Time, Information Workers<br><br>4 cohorts<br><br>1 Year	EMA: Daily for first 56 Days. (Had 4 Hours to Complete)<br><br>Semi-Random Schedule (8AM, Noon, 4PM)	Sent Notifications via Text Message<br><br>Required 7 EMA for inclusion.	Phone: Phone Usage, Locks/Unlocks, GPS<br><br>Watch: (Garmin Vivosmart 3) Heart Rate, Steps, Activity<br><br>Beacons: (with key fob) Home, Work, Weather, Other Participants	Includes Literature Search about Missing Data, Reliability for Stress Studies<br><br>9.5% of the data was missing	EN, RF, FFMLP, GRU, and LSTM.<br><br>Accuracy: 0.19 Spearman correlation = 0.39 Cohen's d
Teh 2022[69]	Mild Cognitive Impairment	49 Participants<br><br>21 with MCI, 28 Healthy<br><br>2 Months	In-Person: Cognitive Assessment		Watch: Heart Rate, Steps<br><br>Home: Motion Sensors, Door Sensors, Bed Sensor<br><br>Beacons: Key and Wallet	Missing data, caused by not wearing watch, beacons<br><br>Fuzzy ARAM model deals with missing values	Fuzzy ARAM<br><br>Accuracy: 58.3% with missing data, 63.6% w/o missing data
Zakaria 2022[123]	Sleep	46 Participants College Students<br><br>4 Weeks<br><br>Additional Smaller Study	In-Person: Sleep Health Assessment<br><br>Additional Study: EMA: Sleep Lop		Wifi: Device usage.<br><br>Wearable: (Oura Ring) Sleep	Wifi: Included all user devices, instead of just smartphone	RF with Infinitesimal Kackknife Variance Estimation<br><br>Accuracy: 5% uncertainty rate
Liang 2023[39]	Stress	10 Participants<br><br>4 weeks	EMA: Every 30 minutes<br><br>Stress-level		Watch: (Garmin) Stress Level (Ground Truth)<br><br>Home: Millimeter Wave Radar to Detect Stress		NN<br><br>Accuracy: 80%
Wyant 2023[50]	Alcohol Use Disorder	154 Participants<br><br>3 Months	EMA: 4/Day (Survey) 4/Day Alcohol Use, Current State/Mood, Exposure to Risks, Sleep<br><br>1/Day (Audio) About Recovery	Initiated Via Text Message	Phone: Location, Call Logs, Text Messages<br><br>Watch: (Empatica E4) Heart Rate, EDA, Temperature<br><br>Home: (Beddit) Sleep Monitor	Quit using watch due to complications and syncing issues.	Measured Acceptability<br><br>Results: Passive methods were more acceptable

[5]Machine-Learning Abbreviations: LR: Linear Regression, LogR: Logistic Regression, DT: Decision Tree, RF: Random Forest, GBM: Gradient Boosting Machines, NB: Naive Bayes, SVM: Support Vector Machine, RF Random Forest, GBRT: Gradient Boosted Regression Trees, XGBRF: XGBoost Random Forest, EN: Elastic Net, MLP: Multi-Layer Perceptron, FFMLP: Feed-Forward Multi-Layer Perceptron, GRU: Gated Recurrent Unit, DEC: Deep Embedded Clustering, MVMDS: Multi-View Multi-Dimensional Scaling, CDMF: Collective Deep Matrix Factorization

Data Availability

The data underlying this article are available in the article and in its online supplementary material.

REFERENCES

1. Shiffman S, Stone AA, Hufford MR. Ecological momentary assessment. Annu Rev Clin Psychol. 2008;4:1-32.
2. Lane ND, Miluzzo E, Lu H, Peebles D, Choudhury T, Campbell AT. A survey of mobile phone sensing. IEEE Communications Magazine. 2010 9;48:140-50.
3. Ságvári B, Gulyás A, Koltai J. Attitudes towards Participation in a Passive Data Collection Experiment. Sensors. 2021 9;21:6085.
4. Maher NA, Senders JT, Hulsbergen AFC, Lamba N, Parker M, Onnela JP, et al. Passive data collection and use in healthcare: A systematic review of ethical issues. International Journal of Medical Informatics. 2019 9;129:242-7.
5. Smets E, Velazquez ER, Schiavone G, Chakroun I, D'Hondt E, Raedt WD, et al. Large-scale wearable data reveal digital phenotypes for daily-life stress detection. npj Digital Medicine 2018 1:1. 2018 12;1:1-10. Available from: https://www.nature.com/articles/s41746-018-0074-9.
6. Ghassemi M, Naumann T, Schulam P, Beam AL, Chen IY, Ranganath R. A Review of Challenges and Opportunities in Machine Learning for Health. AMIA Summits on Translational Science Proceedings. 2020;2020:191. Available from: /pmc/articles/PMC7233077//pmc/articles/PMC7233077/?report=abstracthttps://www.ncbi.nlm.nih.gov/pmc/articles/PMC7233077/.
7. Gashi S, Oldrati P, Moebus M, Hilty M, Barrios L, Ozdemir F, et al. Modeling multiple sclerosis using mobile and wearable sensor data. npj Digital Medicine 2024 7:1. 2024 3;7:1-14. Available from: https://www.nature.com/articles/s41746-024-01025-8.
8. Kargarandehkordi A, Slade C, Washington P. Personalized AI-Driven Real-Time Models to Predict Stress-Induced Blood Pressure Spikes Using Wearable Devices: Proposal for a Prospective Cohort Study. JMIR Research Protocols. 2024 3;13:e55615.
9. Sun Y, Kargarandehkordi A, Slade C, Jaiswal A, Busch G, Guerrero A, et al. Personalized Deep Learning for Substance Use in Hawaii: Protocol for a Passive Sensing and Ecological Momentary Assessment Study. JMIR Res Protoc 2024;13:e46493 https://wwwresearchprotocolsorg/2024/1/e46493. 2024 2;13:e46493. Available from: https://www.researchprotocols.org/2024/1/e46493.
10. Doryab A, Villalba DK, Chikersal P, Dutcher JM, Tumminia M, Liu X, et al. Identifying behavioral phenotypes of loneliness and social isolation with passive sensing: statistical analysis, data mining and machine learning of smartphone and fitbit data. JMIR mHealth and uHealth. 2019;7:e13209.
11. LiKamWa R, Liu Y, Lane ND, Zhong L. Moodscope: Building a mood sensor from smartphone usage patterns. In: Proceeding of the 11th annual international conference on Mobile systems, applications, and services; 2013. p. 389-402.
12. Lind MN, Byrne ML, Wicks G, Smidt AM, Allen NB. The Effortless Assessment of Risk States (EARS) Tool: An Interpersonal Approach to Mobile Sensing. JMIR Ment Health 2018;5(3):e10334 https://mentaljmirorg/2018/3/e10334. 2018 8;5:e10334. Available from: https://mental.jmir.org/2018/3/e10334.
13. Stone AA, Schneider S, Smyth JM. Evaluation of Pressing Issues in Ecological Momentary Assessment. Annual Review of Clinical Psychology. 2023;19.
14. Doherty K, Balaskas A, Doherty G. The design of ecological momentary assessment technologies. Interacting with Computers. 2020;32:257-78.
15. Boonstra TW, Nicholas J, Wong QJJ, Shaw F, Townsend S, Christensen H. Using mobile phone sensor technology for mental health research: integrated analysis to identify hidden challenges and potential solutions. Journal of medical Internet research. 2018;20:e10131.
16. Wang Y, Lin J, Annavaram M, Jacobson QA, Hong J, Krishnamachari B, et al. A framework of energy efficient mobile sensing for automatic user state recognition. MobiSys'09 - Proceedings of the 7th ACM International Conference on Mobile Systems, Applications, and Services. 2009:179-92. Available from: https://dl.acm.org/doi/10.1145/1555816.1555835.
17. Nishiyama Y, Ferreira D, Eigen Y, Sasaki W, Okoshi T, Nakazawa J, et al. IOS crowd–sensing won't hurt a bit!: AWARE framework and sustainable study guideline for iOS platform. In: Distributed, Ambient and Pervasive Interactions: 8th International Conference, DAPI 2020, Held as Part of the 22nd HCI International Conference, HCII 2020, Copenhagen, Denmark, July 19–24, 2020, Proceedings 22; 2020. p. 223-43.
18. Nishiyama Y, Ferreira D, Sasaki W, Okoshi T, Nakazawa J, Dey AK, et al. Using iOS for inconspicuous data collection: A real-world assessment. UbiComp/ISWC 2020 Adjunct - Proceedings of the 2020 ACM International Joint Conference on Pervasive and Ubiquitous Computing and Proceedings of the 2020 ACM International Symposium on Wearable Computers. 2020 9:261-6. Available from: https://dl.acm.org/doi/10.1145/3410530.3414369.
19. Dehling T, Inf DW, Gao F, Schneider S, Sunyaev A. Exploring the Far Side of Mobile Health: Information Security and Privacy of Mobile Health Apps on iOS and Android. JMIR Mhealth Uhealth 2015;3(1):e8 https://mhealthjmirorg/2015/1/e8. 2015 1;3:e3672. Available from: https://mhealth.jmir.org/2015/1/e8.
20. McCurdie T, Taneva S, Casselman M, Yeung M, McDaniel C, Ho W, et al. mHealth consumer apps: the case for user-centered design. Biomedical instrumentation & technology / Association for the Advancement of Medical Instrumentation. 2012;Suppl:49-56.
21. Torous J, Kiang MV, Lorme J, Onnela JP. New Tools for New Research in Psychiatry: A Scalable and Customizable Platform to Empower Data Driven Smartphone Research. JMIR Mental Health. 2016 5;3:e16.
22. Slade C, Benzo RM, Washington P. Design Guidelines for Improving Mobile Sensing Data Collection: Prospective Mixed Methods Study. Journal of Medical Internet Research. 2024 11;26:e55694.

23. Sameh A, Rostami M, Oussalah M, Korpelainen R, Farrahi V. Digital phenotypes and digital biomarkers for health and diseases: a systematic review of machine learning approaches utilizing passive non-invasive signals collected via wearable devices and smartphones. Artificial Intelligence Review. 2024 12;58:66.

24. Seppälä J, Vita ID, Jämsä T, Miettunen J, Isohanni M, Rubinstein K, et al. Mobile Phone and Wearable Sensor-Based mHealth Approaches for Psychiatric Disorders and Symptoms: Systematic Review. JMIR Ment Health 2019;6(2):e9819 https://mentaljmirorg/2019/2/e9819. 2019 2;6:e9819. Available from: `https://mental.jmir.org/2019/2/e9819`.

25. Elwirehardja GN, Isnan M, Perbangsa AS, Muchtar K, Pardamean B. Trends, Opportunities, and Challenges in Detecting Depressive Disorders Through Mobile Devices: A Review. Proceeding - 2023 2nd International Conference on Computer System, Information Technology, and Electrical Engineering: Sustainable Development for Smart Innovation System, COSITE 2023. 2023:188-93.

26. Benoit J, Onyeaka H, Keshavan M, Torous J. Systematic Review of Digital Phenotyping and Machine Learning in Psychosis Spectrum Illnesses. Harvard Review of Psychiatry. 2020 9;28:296-304. Available from: `https://journals.lww.com/hrpjournal/fulltext/2020/09000/systematic_review_of_digital_phenotyping_and.2.aspx`.

27. Teepe GW, da Fonseca A, Kleim B, Jacobson NC, Sanabria AS, Car LT, et al. Just-in-Time Adaptive Mechanisms of Popular Mobile Apps for Individuals With Depression: Systematic App Search and Literature Review. J Med Internet Res 2021;23(9):e29412 https://wwwjmirorg/2021/9/e29412. 2021 9;23:e29412. Available from: `https://www.jmir.org/2021/9/e29412`.

28. Thieme A, Belgrave D, Doherty G. Machine Learning in Mental Health: A Systematic Review of the HCI Literature to Support the Development of Effective and Implementable ML Systems. ACM Transactions on Computer-Human Interaction. 2020 10;27:1-53.

29. Saganowski S, Perz B, Polak AG, Kazienko P. Emotion Recognition for Everyday Life Using Physiological Signals From Wearables: A Systematic Literature Review. IEEE Transactions on Affective Computing. 2023 7;14:1876-97.

30. Assabumrungrat R, Sangnark S, Charoenpattarawut T, Polpakdee W, Sudhawiyangkul T, Boonchieng E, et al. Ubiquitous Affective Computing: A Review. IEEE Sensors Journal. 2022 2;22:1867-81.

31. Bota PJ, Wang C, Fred ALN, Silva HPD. A Review, Current Challenges, and Future Possibilities on Emotion Recognition Using Machine Learning and Physiological Signals. IEEE Access. 2019;7:140990-1020.

32. Hussain Z, Sheng QZ, Zhang WE, Ortiz J, Pouriyeh S. Non-invasive Techniques for Monitoring Different Aspects of Sleep: A Comprehensive Review. ACM Transactions on Computing for Healthcare (HEALTH). 2022 3;3. Available from: `https://dl.acm.org/doi/10.1145/3491245`.

33. Saboor A, Kask T, Kuusik A, Alam MM, Moullec YL, Niazi IK, et al. Latest research trends in gait analysis using wearable sensors and machine learning: A systematic review. IEEE Access. 2020;8:167830-64.

34. Stuijt DG, Radanovic I, Kos M, Schoones JW, Stuurman FE, Exadaktylos V, et al. Smartphone-Based Passive Sensing in Monitoring Patients With Cancer: A Systematic Review. JCO Clinical Cancer Informatics. 2023 9. Available from: `https://ascopubs.org/doi/10.1200/CCI.23.00141`.

35. Kumar D, Jeuris S, Bardram JE, Dragoni N. Mobile and Wearable Sensing Frameworks for mHealth Studies and Applications. ACM Transactions on Computing for Healthcare. 2020 12;2. Available from: `https://dl.acm.org/doi/10.1145/3422158`.

36. Trifan A, Oliveira M, Oliveira JL. Passive Sensing of Health Outcomes Through Smartphones: Systematic Review of Current Solutions and Possible Limitations. JMIR Mhealth Uhealth 2019;7(8):e12649 https://mhealthjmirorg/2019/8/e12649. 2019 8;7:e12649. Available from: `https://mhealth.jmir.org/2019/8/e12649`.

37. Kulkarni P, Kirkham R, McNaney R. Opportunities for Smartphone Sensing in E-Health Research: A Narrative Review. Sensors. 2022 5;22:3893.

38. Peters MDJ, Marnie C, Tricco AC, Pollock D, Munn Z, Alexander L, et al. Updated methodological guidance for the conduct of scoping reviews. JBI Evidence Synthesis. 2020 10;18:2119-26.

39. Liang K, Zhou A, Zhang Z, Zhou H, Ma H, Wu C. mmStress: Distilling Human Stress from Daily Activities via Contactless Millimeter-wave Sensing. Proceedings of the ACM on Interactive, Mobile, Wearable and Ubiquitous Technologies. 2023 9;7:1-36. Available from: `https://dl.acm.org/doi/10.1145/3610926`.

40. Tsai CH, Chen PC, Liu DS, Ying-Ying TTK, Hsieh, Chiang DL, et al. Panic Attack Prediction Using Wearable Devices and Machine Learning: Development and Cohort Study. JMIR MEDICAL INFORMATICS. 2022 2;10.

41. Presseller EK, Parker MN, Zhang F, Manasse S, Juarascio AS. Continuous glucose monitoring as an objective measure of meal consumption in individuals with binge-spectrum eating disorders: A proof-of-concept study. EUROPEAN EATING DISORDERS REVIEW. 2024 5.

42. Hafiz P, Miskowiak KW, Maxhuni A, Kessing LV, Bardram JE. Wearable Computing Technology for Assessment of Cognitive Functioning of Bipolar Patients and Healthy Controls. Proceedings of the ACM on Interactive, Mobile, Wearable and Ubiquitous Technologies. 2020 12;4:1-22. Available from: `https://dl.acm.org/doi/10.1145/3432219`.

43. Chen R, Jankovic F, Marinsek N, Foschini L, Kourtis L, Signorini A, et al. Developing Measures of Cognitive Impairment in the Real World from Consumer-Grade Multimodal Sensor Streams. In: Proceedings of the 25th ACM SIGKDD International Conference on Knowledge Discovery & Data Mining. ACM; 2019. p. 2145-55. Available from: `https://dl.acm.org/doi/10.1145/3292500.3330690`.

44. Chatterjee S, Moreno A, Lizotte SL, Akther S, Ertin E, Fagundes CP, et al. SmokingOpp: Detecting the Smoking 'Opportunity' Context Using Mobile Sensors. Proceedings of the ACM on Interactive, Mobile, Wearable and Ubiquitous Technologies. 2020 3;4:1-26. Available from: `https://dl.acm.org/doi/10.1145/3380987`.

45. Tong C, Craner M, Vegreville M, Lane ND. Tracking Fatigue and Health State in Multiple Sclerosis Patients Using Connnected Wellness Devices. Proceedings of the ACM on Interactive, Mobile, Wearable and Ubiquitous Technologies.

2019 9;3:1-19. Available from: https://dl.acm.org/doi/10.1145/3351264.

46. Booth BM, Vrzakova H, Mattingly SM, Martinez GJ, Faust L, D'Mello SK. Toward Robust Stress Prediction in the Age of Wearables: Modeling Perceived Stress in a Longitudinal Study With Information Workers. IEEE Transactions on Affective Computing. 2022 10;13:2201-17. Available from: https://ieeexplore.ieee.org/document/9813544/.

47. CAN YS, ERSOY C. Smart Affect Monitoring With Wearables in the Wild: An Unobtrusive Mood-Aware Emotion Recognition System. IEEE Transactions on Affective Computing. 2023 10;14:2851-63. Available from: https://ieeexplore.ieee.org/document/9999489/.

48. Adler DA, Tseng VWS, Qi G, Scarpa J, Sen S, Choudhury T. Identifying Mobile Sensing Indicators of Stress-Resilience. Proceedings of the ACM on Interactive, Mobile, Wearable and Ubiquitous Technologies. 2021 6;5:1-32. Available from: https://dl.acm.org/doi/10.1145/3463528.

49. Wang R, Aung MSH, Abdullah S, Brian R, Campbell AT, Choudhury T, et al. CrossCheck: toward passive sensing and detection of mental health changes in people with schizophrenia. In: Proceedings of the 2016 ACM International Joint Conference on Pervasive and Ubiquitous Computing. ACM; 2016. p. 886-97.

50. Wyant K, Moshontz H, Ward SB, Fronk GE, Curtin JJ. Acceptability of Personal Sensing Among People With Alcohol Use Disorder: Observational Study. JMIR MHEALTH AND UHEALTH. 2023;11.

51. King ZD, Yu H, Vaessen T, Inez AMG, Sano. Investigating Receptivity and Affect Using Machine Learning: Ecological Momentary Assessment and Wearable Sensing Study. JMIR MHEALTH AND UHEALTH. 2024;12.

52. Bae S, Chung T, Ferreira D, Dey AK, Suffoletto B. Mobile phone sensors and supervised machine learning to identify alcohol use events in young adults: Implications for just-in-time adaptive interventions. ADDICTIVE BEHAVIORS. 2018 8;83:42-7.

53. Kim T, Kim H, Lee HY, Goh H, Abdigapporov S, Jeong M, et al. Prediction for Retrospection: Integrating Algorithmic Stress Prediction into Personal Informatics Systems for College Students' Mental Health. Conference on Human Factors in Computing Systems - Proceedings. 2022 4. Available from: https://dl-acm-org.eres.library.manoa.hawaii.edu/doi/10.1145/3491102.3517701.

54. Jacobson NC, Chung YJ. Passive Sensing of Prediction of Moment-To-Moment Depressed Mood among Undergraduates with Clinical Levels of Depression Sample Using Smartphones. SENSORS. 2020 6;20.

55. Theilig MM, Korbel JJ, Mayer G, Hoffmann C, Zarnekow R. Employing Environmental Data and Machine Learning to Improve Mobile Health Receptivity. IEEE Access. 2019;7:179823-41.

56. Luštrek M, Lukan J, Bolliger L, Lauwerier E, Clays E. Designing an Intervention against Occupational Stress Based on Ubiquitous Stress and Context Detection. In: Adjunct Proceedings of the 2023 ACM International Joint Conference on Pervasive and Ubiquitous Computing & the 2023 ACM International Symposium on Wearable Computing. ACM; 2023. p. 606-10. Available from: https://dl.acm.org/doi/10.1145/3594739.3611326.

57. King ZD, Moskowitz J, Egilmez B, Zhang S, Zhang L, Bass M, et al. micro-Stress EMA: A Passive Sensing Framework for Detecting in-the-wild Stress in Pregnant Mothers. Proceedings of the ACM on Interactive, Mobile, Wearable and Ubiquitous Technologies. 2019 9;3:1-22. Available from: https://dl-acm-org.eres.library.manoa.hawaii.edu/doi/10.1145/3351249.

58. Arakawa R, Ahuja K, Mak K, Thompson G, Shaaban S, Lindhiem O, et al. LemurDx: Using Unconstrained Passive Sensing for an Objective Measurement of Hyperactivity in Children with no Parent Input. Proceedings of the ACM on Interactive, Mobile, Wearable and Ubiquitous Technologies. 2023 6;7:1-23. Available from: https://dl.acm.org/doi/10.1145/3596244.

59. Kao HT, Yan S, Hosseinmardi H, Narayanan S, Lerman K, Ferrara E. User-Based Collaborative Filtering Mobile Health System. Proceedings of the ACM on Interactive, Mobile, Wearable and Ubiquitous Technologies. 2020 12;4:1-17. Available from: https://dl.acm.org/doi/10.1145/3432703.

60. Kumar D, Maharjan R, Maxhuni A, Dominguez H, Frølich A, Bardram JE. mCardia: A Context-Aware ECG Collection System for Ambulatory Arrhythmia Screening. ACM Transactions on Computing for Healthcare. 2022 4;3:1-28. Available from: https://dl.acm.org/doi/10.1145/3494581.

61. Lee J, Kim S, Cheon M, Ju H, Lee J, Hwang I. SleepGuru: Personalized Sleep Planning System for Real-life Actionability and Negotiability. In: Proceedings of the 35th Annual ACM Symposium on User Interface Software and Technology. ACM; 2022. p. 1-16. Available from: https://dl.acm.org/doi/10.1145/3526113.3545709.

62. Kunchay S, Abdullah S. WatchOver: Using Apple watches to assess and predict substance co-use in young adults. UbiComp/ISWC 2020 Adjunct - Proceedings of the 2020 ACM International Joint Conference on Pervasive and Ubiquitous Computing and Proceedings of the 2020 ACM International Symposium on Wearable Computers. 2020 9:488-93. Available from: https://dl-acm-org.eres.library.manoa.hawaii.edu/doi/10.1145/3410530.3414373.

63. Saha K, Chan L, Barbaro KD, Abowd GD, Choudhury MD. Inferring Mood Instability on Social Media by Leveraging Ecological Momentary Assessments. Proceedings of the ACM on Interactive, Mobile, Wearable and Ubiquitous Technologies. 2017 9;1:1-27. Available from: https://dl.acm.org/doi/10.1145/3130960.

64. Buda TS, Khwaja M, Matic A. Outliers in Smartphone Sensor Data Reveal Outliers in Daily Happiness. Proceedings of the ACM on Interactive, Mobile, Wearable and Ubiquitous Technologies. 2021 3;5:1-19. Available from: https://dl.acm.org/doi/10.1145/3448095.

65. Srikanthan S, Asani F, Patel BK, Agu E. Smartphone TBI Sensing using Deep Embedded Clustering and Extreme Boosted Outlier Detection. In: 2021 IEEE International Conference on Digital Health (ICDH). IEEE; 2021. p. 122-32. Available from: https://ieeexplore.ieee.org/document/9581222/.

66. Zhang X, Zhuang F, Li W, Ying H, Xiong H, Lu S. Inferring Mood Instability via Smartphone Sensing: A Multi-View Learning Approach. In: Proceedings of the 27th ACM International Conference on Multimedia. ACM; 2019. p. 1401-9. Available from: https://dl.acm.org/doi/10.1145/3343031.3350957.

67. Singh VK, Goyal R, Wu S. Riskalyzer: Inferring Individual Risk-Taking Propensity Using Phone Metadata. Proceedings of the ACM on Interactive, Mobile, Wearable and Ubiquitous Technologies. 2018 3;2:1-21. Available from: https://dl.acm.org/doi/10.1145/3191766.

68. Rhim S, Lee U, Han K. Tracking and Modeling Subjective Well-Being Using Smartphone-Based Digital Phenotype. In: Proceedings of the 28th ACM Conference on User Modeling, Adaptation and Personalization. ACM; 2020. p. 211-20. Available from: https://dl.acm.org/doi/10.1145/3340631.3394855.

69. Teh SK, Rawtaer I, Tan AH. Predictive self-organizing neural networks for in-home detection of MildCognitive Impairment. EXPERT SYSTEMS WITH APPLICATIONS. 2022 11;205.

70. Cook DJ, Schmitter-Edgecombe M. Fusing Ambient and Mobile Sensor Features Into a Behaviorome for Predicting Clinical Health Scores. IEEE Access. 2021;9:65033-43. Available from: https://ieeexplore.ieee.org/document/9417180/.

71. Zhang S, Zhao Y, Nguyen DT, Xu R, Sen S, Hester J, et al. NeckSense: A Multi-Sensor Necklace for Detecting Eating Activities in Free-Living Conditions. Proceedings of the ACM on Interactive, Mobile, Wearable and Ubiquitous Technologies. 2020 6;4:1-26. Available from: https://dl.acm.org/doi/10.1145/3397313.

72. Nepal S, Liu W, Pillai A, Wang W, Vojdanovski V, Huckins JF, et al. Capturing the College Experience: A Four-Year Mobile Sensing Study of Mental Health, Resilience and Behavior of College Students during the Pandemic. Proceedings of the ACM on Interactive, Mobile, Wearable and Ubiquitous Technologies. 2024 3;8:1-37. Available from: https://dl.acm.org/doi/10.1145/3643501.

73. Wang W, Nepal S, Huckins JF, Hernandez L, Vojdanovski V, Mack D, et al. First-gen lens: Assessing mental health of first-generation students across their first year at college using mobile sensing. Proceedings of the ACM on Interactive, Mobile, Wearable and Ubiquitous Technologies. 2022 7;6:1-32. Available from: https://dl.acm.org/doi/10.1145/3543194.

74. Biel JI, Martin N, Labbe D, Gatica-Perez D. Bites'n'Bits: Inferring Eating Behavior from Contextual Mobile Data. Proceedings of the ACM on Interactive, Mobile, Wearable and Ubiquitous Technologies. 2018 1;1:1-33. Available from: https://dl.acm.org/doi/10.1145/3161161.

75. Rashid H, Mendu S, Daniel KE, Beltzer ML, Teachman BA, Boukhechba M, et al. Predicting Subjective Measures of Social Anxiety from Sparsely Collected Mobile Sensor Data. Proceedings of the ACM on Interactive, Mobile, Wearable and Ubiquitous Technologies. 2020 9;4:109. Available from: https://dl-acm-org.eres.library.manoa.hawaii.edu/doi/10.1145/3411823.

76. Baglione AN, Gong J, Boukhechba M, Wells KJ, Barnes LE. Leveraging Mobile Sensing to Understand and Develop Intervention Strategies to Improve Medication Adherence. IEEE Pervasive Computing. 2020 7;19:24-36. Available from: https://ieeexplore.ieee.org/document/9127191/.

77. Velmovitsky PE, Alencar P, Leatherdale ST, Cowan D, Morita PP. A Novel Mobile Platform for Stress Prediction: Application, Protocol and Preliminary Results. DigiBiom 2022 - Proceedings of the 2022 Emerging Devices for Digital Biomarkers. 2022 7:18-23. Available from: https://dl-acm-org.eres.library.manoa.hawaii.edu/doi/10.1145/3539494.3542752.

78. Bardram JE, Cramer-Petersen C, Maxhuni A, Christensen MVS, Bækgaard P, Persson DR, et al. DiaFocus: A Personal Health Technology for Adaptive Assessment in Long-Term Management of Type 2 Diabetes. ACM Transactions on Computing for Healthcare. 2023 4;4:1-43. Available from: https://dl.acm.org/doi/10.1145/3586579.

79. Zhou M, Fukuoka Y, Mintz Y, Goldberg K, Kaminsky P, Flowers E, et al. Evaluating Machine Learning-Based Automated Personalized Daily Step Goals Delivered Through a Mobile Phone App: Randomized Controlled Trial. JMIR MHEALTH AND UHEALTH. 2018 1;6.

80. Use Focus on your iPhone or iPad - Apple Support;. Accessed: 2023-11-22. https://support.apple.com/en-us/HT212608.

81. Pais D. Get into "Focus mode" at work with help from Android;. Accessed: 2023-11-22. https://blog.google/products/android-enterprise/focus-mode/#.

82. Auda J, Weber D, Voit A, Schneegass S. Understanding user preferences towards rule-based notification deferral. In: Extended Abstracts of the 2018 CHI Conference on Human Factors in Computing Systems; 2018. p. 1-6.

83. Li T, Haines JK, Eguino MFRD, Hong JI, Nichols J. Alert Now or Never: Understanding and Predicting Notification Preferences of Smartphone Users. ACM Transactions on Computer-Human Interaction. 2023;29:1-33.

84. Torkamaan H, Ziegler J. Mobile Mood Tracking: An Investigation of Concise and Adaptive Measurement Instruments. Proceedings of the ACM on Interactive, Mobile, Wearable and Ubiquitous Technologies. 2020 12;4:1-30. Available from: https://dl.acm.org/doi/10.1145/3432207.

85. Chang X, Peng C, Xing G, Hao T, Zhou G. iSleep: A Smartphone System for Unobtrusive Sleep Quality Monitoring. ACM Transactions on Sensor Networks. 2020 8;16:1-32. Available from: https://dl.acm.org/doi/10.1145/3392049.

86. Mishra V, Pope G, Lord S, Lewia S, Lowens B, Caine K, et al. Continuous Detection of Physiological Stress with Commodity Hardware. ACM Transactions on Computing for Healthcare. 2020 4;1. Available from: https://dl.acm.org/doi/10.1145/3361562.

87. Morrison LG, Hargood C, Pejovic V, Geraghty AWA, Lloyd S, Goodman N, et al. The Effect of Timing and Frequency of Push Notifications on Usage of a Smartphone-Based Stress Management Intervention: An Exploratory Trial. PLOS ONE. 2017 1;12:e0169162. Available from: https://journals.plos.org/plosone/article?id=10.1371/journal.pone.0169162.

88. Morshed MB, Saha K, Li R, D'Mello SK, Choudhury MD, Abowd GD, et al. Prediction of Mood Instability with Passive Sensing. Proceedings of the ACM on Interactive, Mobile, Wearable and Ubiquitous Technologies. 2019 9;3:1-21. Available from: https://dl-acm-org.eres.library.manoa.hawaii.edu/doi/10.1145/3351233.

89. Aung MSH, Alquaddoomi F, Hsieh CK, Rabbi M, Yang L, Pollak JP, et al. Leveraging Multi-Modal Sensing for Mobile Health: A Case Review in Chronic Pain. IEEE Journal of Selected Topics in Signal Processing. 2016 8;10:962-74.

90. Huang Y, Xiong H, Leach K, Zhang Y, Chow P, Fua K, et al. Assessing social anxiety using GPS trajectories and point-of-interest data. UbiComp 2016 - Proceedings of the 2016 ACM International Joint Conference on Pervasive and Ubiquitous

Computing. 2016 9:898-903. Available from: https://dl.acm.org/doi/10.1145/2971648.2971761.

91. DeMasi O, Recht B. A step towards quantifying when an algorithm can and cannot predict an individual's wellbeing. UbiComp/ISWC 2017 - Adjunct Proceedings of the 2017 ACM International Joint Conference on Pervasive and Ubiquitous Computing and Proceedings of the 2017 ACM International Symposium on Wearable Computers. 2017 9:763-71. Available from: https://dl-acm-org.eres.library.manoa.hawaii.edu/doi/10.1145/3123024.3125609.

92. Spanakis G, Weiss G, Boh B, Lemmens L, Roefs A. Machine learning techniques in eating behavior e-coaching: Balancing between generalization and personalization. Personal and Ubiquitous Computing. 2017 8;21:645-59. Available from: https://link-springer-com.eres.library.manoa.hawaii.edu/article/10.1007/s00779-017-1022-4.

93. Cai L, Boukhechba M, Wu C, Chow PI, Teachman BA, Barnes LE, et al. State affect recognition using smartphone sensing data. In: Proceedings of the 2018 IEEE/ACM International Conference on Connected Health: Applications, Systems and Engineering Technologies. ACM; 2018. p. 120-5. Available from: https://dl.acm.org/doi/10.1145/3278576.3284386.

94. Wang R, Wang W, Aung MH, Ben-Zeev D, Brian R, Campbell AT, et al. Predicting Symptom Trajectories of Schizophrenia Using Mobile Sensing. GetMobile: Mobile Computing and Communications. 2018 9;22:32-7. Available from: https://dl-acm-org.eres.library.manoa.hawaii.edu/doi/10.1145/3276145.3276157.

95. Etienne N, Agu E. Investigating Transfer Learning of Smartphone-Sensed Stress in University Populations. Proceedings - 2020 IEEE International Conference on Big Data, Big Data 2020. 2020 12:4850-8.

96. Ameko MK, Beltzer ML, Cai L, Boukhechba M, Teachman BA, Barnes LE. Offline Contextual Multi-armed Bandits for Mobile Health Interventions: A Case Study on Emotion Regulation. In: Fourteenth ACM Conference on Recommender Systems. ACM; 2020. p. 249-58. Available from: https://dl.acm.org/doi/10.1145/3383313.3412244.

97. Obuchi M, Huckins JF, Wang W, Dasilva A, Rogers C, Murphy E, et al. Predicting Brain Functional Connectivity Using Mobile Sensing. Proceedings of the ACM on Interactive, Mobile, Wearable and Ubiquitous Technologies. 2020 3;4:22. Available from: https://dl-acm-org.eres.library.manoa.hawaii.edu/doi/10.1145/3381001.

98. Dong G, Cai L, Datta D, Kumar S, Barnes LE, Boukhechba M. Influenza-like symptom recognition using mobile sensing and graph neural networks. In: Proceedings of the Conference on Health, Inference, and Learning. vol. 21. ACM; 2021. p. 291-300. Available from: https://dl.acm.org/doi/10.1145/3450439.3451880.

99. Dong G, Tang M, Cai L, Barnes LE, Boukhechba M. Semi-supervised Graph Instance Transformer for Mental Health Inference. In: 2021 20th IEEE International Conference on Machine Learning and Applications (ICMLA). IEEE; 2021. p. 1221-8. Available from: https://ieeexplore.ieee.org/document/9679981/.

100. Meegahapola L, Ruiz-Correa S, del Carmen Robledo-Valero V, Hernandez-Huerfano EE, Alvarez-Rivera L, Chenu-Abente R, et al. One More Bite? Inferring Food Consumption Level of College Students Using Smartphone Sensing and Self-Reports. Proceedings of the ACM on Interactive, Mobile, Wearable and Ubiquitous Technologies. 2021 3;5:1-28. Available from: https://dl.acm.org/doi/10.1145/3448120.

101. Meegahapola L, Labhart F, Phan TT, Gatica-Perez D. Examining the Social Context of Alcohol Drinking in Young Adults with Smartphone Sensing. Proceedings of the ACM on Interactive, Mobile, Wearable and Ubiquitous Technologies. 2021 9;5:1-26. Available from: https://dl.acm.org/doi/10.1145/3478126.

102. Meegahapola L, Droz W, Kun P, de Götzen A, Nutakki C, Diwakar S, et al. Generalization and Personalization of Mobile Sensing-Based Mood Inference Models. Proceedings of the ACM on Interactive, Mobile, Wearable and Ubiquitous Technologies. 2022 12;6:1-32. Available from: https://dl.acm.org/doi/10.1145/3569483.

103. Bae SW, Suffoletto B, Zhang T, Tammy MC, Ozolcer, Islam MR, et al. Leveraging Mobile Phone Sensors, Machine Learning, and Explainable Artificial Intelligence to Predict Imminent Same-Day Binge-drinking Events to Support Just-in-time Adaptive Interventions: Algorithm Development and Validation Study. JMIR FORMATIVE RESEARCH. 2023;7.

104. Ning E, Cladek AT, Ross MK, Kabir S, Barve A, Kennelly E, et al. Smartphone-derived Virtual Keyboard Dynamics Coupled with Accelerometer Data as a Window into Understanding Brain Health: Smartphone Keyboard and Accelerometer as Window into Brain Health. Conference on Human Factors in Computing Systems - Proceedings. 2023 4;15. Available from: https://dl-acm-org.eres.library.manoa.hawaii.edu/doi/10.1145/3544548.3580906.

105. Vandelanotte C, Trost S, Hodgetts D, Imam T, Rashid M, To QG, et al. Increasing physical activity using an just-in-time adaptive digital assistant supported by machine learning: A novel approach for hyper-personalised mHealth interventions. JOURNAL OF BIOMEDICAL INFORMATICS. 2023 8;144.

106. Wang Z, Larrazabal MA, Rucker M, Toner ER, Daniel KE, Kumar S, et al. Detecting social contexts from mobile sensing indicators in virtual interactions with socially anxious individuals. Proceedings of the ACM on Interactive, Mobile, Wearable and Ubiquitous Technologies. 2023;7:1-26.

107. Gjoreski M, Gjoreski H, Luštrek M, Gams M. Continuous stress detection using a wrist device - in laboratory and real life. UbiComp 2016 Adjunct - Proceedings of the 2016 ACM International Joint Conference on Pervasive and Ubiquitous Computing. 2016 9:1185-93. Available from: https://dl.acm.org/doi/10.1145/2968219.2968306.

108. Sefidgar YS, Seo W, Kuehn KS, Althoff T, Browning A, Riskin E, et al. Passively-sensed Behavioral Correlates of Discrimination Events in College Students. Proceedings of the ACM on Human-Computer Interaction. 2019 11;3. Available from: https://dl.acm.org/doi/10.1145/3359216.

109. Bonaquist A, Grehan M, Haines O, Keogh J, Mullick T, Singh N, et al. An Automated Machine Learning Pipeline for Monitoring and Forecasting Mobile Health Data. 2021 IEEE Systems and Information Engineering Design Symposium, SIEDS 2021. 2021 4.

110. Wang W, Xu W, Chander A, Nepal S, Buck B, Pakhomov S, et al. The Power of Speech in the Wild: Discriminative Power of Daily Voice Diaries in Understanding Auditory Verbal Hallucinations Using Deep Learning. Proceedings of the ACM on Interactive, Mobile, Wearable and Ubiquitous Technologies. 2023 9;7:29. Available from: https://doi.org/10.1145/3610890.

111. Toshnazarov K, Lee U, Kim BH, Mishra V, Najarro LAC, Noh Y. SOSW: Stress Sensing With Off-the-Shelf Smartwatches in the Wild. IEEE Internet of Things Journal. 2024.

112. Chatterjee S, Hovsepianr K, Sarker H, Saleheen N, Al'absiy M, Atluriy G, et al. mCrave: Continuous estimation of craving during smoking cessation. UbiComp 2016 - Proceedings of the 2016 ACM International Joint Conference on Pervasive and Ubiquitous Computing. 2016 9:863-74. Available from: https://dl.acm.org/doi/10.1145/2971648.2971672.

113. Sarker H, Tyburskir M, Rahman MDM, Hovsepian K, Sharmin M, Epsteinr DH, et al. Finding significant stress episodes in a discontinuous time series of rapidly varying mobile sensor data. Conference on Human Factors in Computing Systems - Proceedings. 2016 5:4489-501. Available from: https://dl.acm.org/doi/10.1145/2858036.2858218.

114. Delmastro F, Martino FD, Dolciotti C. Cognitive Training and Stress Detection in MCI Frail Older People Through Wearable Sensors and Machine Learning. IEEE Access. 2020;8:65573-90. Available from: https://ieeexplore.ieee.org/document/9055213/.

115. Gupta K, Gujral A, Chaspari T, Timmons AC, Han S, Kim Y, et al. Sub-Population Specific Models of Couples' Conflict. ACM Transactions on Internet Technology. 2020 5;20:1-20. Available from: https://dl.acm.org/doi/10.1145/3372045.

116. Elvitigala DS, Scholl PM, Suriyaarachchi H, DIssanayake V, Nanayakkara S. StressShoe: A DIY Toolkit for just-in-time Personalised Stress Interventions for Office Workers Performing Sedentary Tasks. Proceedings of MobileHCI 2021 - ACM International Conference on Mobile Human-Computer Interaction: Mobile Apart, MobileTogether. 2021 9. Available from: https://dl-acm-org.eres.library.manoa.hawaii.edu/doi/10.1145/3447526.3472023.

117. Ullah MA, Chatterjee S, Fagundes CP, Lam C, Nahum-Shani I, Rehg JM, et al. mRisk: Continuous Risk Estimation for Smoking Lapse from Noisy Sensor Data with Incomplete and Positive-Only Labels. Proceedings of the ACM on Interactive, Mobile, Wearable and Ubiquitous Technologies. 2022 9;6:1-29. Available from: https://dl.acm.org/doi/10.1145/3550308.

118. Daryabeygi-Khotbehsara R, Rawstorn JC, Dunstan DW, Islam SMS, Abdelrazek M, Kouzani AZ, et al. A Bluetooth-Enabled Device for Real-Time Detection of Sitting, Standing, and Walking: Cross-Sectional Validation Study. JMIR FORMATIVE RESEARCH. 2024;8.

119. Diethe T, Nieto MP, Tonkin E, Holmes M, Sokol K, Twomey N, et al. Releasing eHealth analytics into the wild: Lessons learnt from the SPHERE project. Proceedings of the ACM SIGKDD International Conference on Knowledge Discovery and Data Mining. 2018 7:243-52. Available from: https://dl.acm.org/doi/10.1145/3219819.3219883.

120. Goldstein SP, Thomas JG, Foster GD, Turner-McGrievy G, Butryn ML, Herbert JD, et al. Refining an algorithm-powered just-in-time adaptive weight control intervention: A randomized controlled trial evaluating model performance and behavioral outcomes. Health Informatics Journal. 2020 12;26:2315-31. Available from: https://journals-sagepub-com.eres.library.manoa.hawaii.edu/doi/10.1177/1460458220902330.

121. Saha K, Grover T, Mattingly SM, swain VD, Gupta P, Martinez GJ, et al. Person-Centered Predictions of Psychological Constructs with Social Media Contextualized by Multimodal Sensing. Proceedings of the ACM on Interactive, Mobile, Wearable and Ubiquitous Technologies. 2021 3;5:1-32. Available from: https://dl.acm.org/doi/10.1145/3448117.

122. Shin J, Moon J, Kim B, Eom J, Park N, Lee K. Attention-based stress detection exploiting non-contact monitoring of movement patterns with IR-UWB radar. Proceedings of the ACM Symposium on Applied Computing. 2021 3:637-40. Available from: https://dl.acm.org/doi/10.1145/3412841.3442089.

123. Zakaria C, Yilmaz G, Mammen PM, Chee M, Shenoy P, Balan R. SleepMore: Inferring Sleep Duration at Scale via Multi-Device WiFi Sensing. Proceedings of the ACM on Interactive, Mobile, Wearable and Ubiquitous Technologies. 2022 12;6:1-32. Available from: https://dl.acm.org/doi/10.1145/3569489.
